# Use of ‘Pharmaceutical services’ Medical Subject Headings (MeSH) in articles assessing pharmacists' interventions

**DOI:** 10.1016/j.rcsop.2022.100172

**Published:** 2022-08-20

**Authors:** Fernanda S. Tonin, Vanessa Gmünder, Aline F. Bonetti, Antonio M. Mendes, Fernando Fernandez-Llimos

**Affiliations:** a H&TRC - Health & Technology Research Center, ESTeSL - Escola Superior de Tecnologia da Saúde, Instituto Politécnico de Lisboa, Lisbon, Portugal; b Pharmaceutical Care, Department of Pharmaceutical Sciences, University of Basel, Basel, Switzerland; c Pharmaceutical Sciences Postgraduate Research Program, Federal University of Paraná, Curitiba, Brazil; d Pharmacy Service, Hospital de Clínicas, Federal University of Paraná, Curitiba, Brazil; e Laboratory of Pharmacology, Department of Drug Sciences, Faculty of Pharmacy, University of Porto, Porto, Portugal; f Center for Health Technology and Services Research (CINTESIS), University of Porto, Porto, Portugal

**Keywords:** Pharmaceutical services, Pharmacists, Medical subject headings, Bibliometrics, Periodicals as topic, MEDLINE

## Abstract

**Background:**

Medical Subject Headings (MeSH) thesaurus contribute towards efficient searching of biomedical information. However, insufficient coverage of specific fields and inaccuracies in the indexing of articles can lead to bias during literature retrieval.

**Objectives:**

This meta-research study aimed to assess the use of ‘Pharmaceutical Services’ MeSH terms in studies evaluating the effect of pharmacists' interventions.

**Methods:**

An updated systematic search (Jan-2022) to gather meta-analyses comparing pharmacists' interventions vs. other forms of care was performed. All MeSH terms allocated to the MEDLINE record of each primary study included in the selected meta-analyses were systematically extracted. Terms from the ‘Pharmaceutical Services’ branch, including its descendants, as well as other 26 pharmacy-specific MeSH terms were identified. The assignment of these terms as a ‘Major MeSH’ was also evaluated. Descriptive statistics and social network analyses to evaluate the co-occurrence of the MeSH terms in the articles were conducted. Sensitivity analyses including only meta-analyses with declared objectives mentioning the words ‘pharmacist’ or ‘pharmacy’ were performed (SPSS v.24.0).

**Results:**

Overall, 138 meta-analyses including 2012 primary articles were evaluated. A median of 15 [IQR 12–18] MeSH terms were assigned per article with a slight positive time-trend (Spearman rho = 0.193; *p* < 0.001). Only 36.6% (*n* = 736/2012) and 58.1% (*n* = 338/1099) of studies were indexed with one MeSH term from the ‘Pharmaceutical Services’ branch in the overall and sensitivity analyses, respectively. In <20% of cases, these terms were a ‘Major MeSH’. The pharmacy-specific term ‘Pharmacists’ was the most frequently used, yet in only 27.8% and 47.7% of articles in the original and sensitivity analyses, respectively. Social networks showed a weak association between pharmacy-specific and ‘Pharmaceutical services’ branch MeSH terms.

**Conclusions:**

The availability of a ‘Pharmaceutical services’ branch hierarchic tree and further pharmacy-specific MeSH terms incorporated to the MeSH thesaurus in the past years is not related with accurate indexing of articles.

## Highlights

• ‘Pharmaceutical Services’ MeSH terms are poorly assigned to pharmacist interventions articles.
• Among the 26 pharmacy-specific MeSH terms, only ‘Pharmacists’ is fairly used.
• There is a weak co-occurrence of pharmacy MeSH terms in pharmacy practice articles.

## 1 Introduction

Medical Subject Headings (MeSH) thesaurus is the controlled vocabulary created by the U.S. National Library of Medicine's (NLM) to index and catalog different biomedical sources of information (e.g., articles, books). This thesaurus was created in 1960's, comprising about 4000 terms, as the evolution of the subject headings printed on the dividers used in library card cabinets. In 2021, the number of descriptors almost reached 30,000.^1^ MeSH thesaurus is organized in a hierarchic structure, with terms describing broader concepts upper in the tree structure, with descendent MeSH terms describing narrower (i.e., more specific) concepts.

Probably, the most important utility for researchers of MeSH thesaurus is its contribution to more efficient literature searches. MEDLINE, one of the databases included in PubMed, comprises >28 million records of the 32 million existing in PubMed, having all of them MeSH terms assigned by the NLM staff or subcontracted catalogers.^2^ Previous studies demonstrated that the use of MeSH terms significantly facilitates the retrieval of relevant articles when compared to the use of text words, especially when variant terminologies around the same topic exist.^3^, ^4^, ^5^, ^6^

Despite the apparent comprehensiveness of the MeSH thesaurus covering all biomedical areas, studies show that the coverage of specific fields is insufficient.^7^^,^^8^ Minguet et al. (2013) identified only 26 pharmacy-specific MeSH terms available, compared to the 145 and 94 figures for the fields of dentistry and nursing, respectively.^9^ Subsequently, these authors suggested 16 new MeSH terms to better characterize the pharmacy practice area. Five of the suggested MeSH were then created in yearly updates.^10^^,^^11^

However, enhancing the coverage of an area by MeSH thesaurus is not sufficient. Existing MeSH terms should be appropriately assigned to articles by NLM indexers. MeSH selection for pharmacy practice articles was also criticized. Minguet et al. (2015) evaluated the MeSH assignment to articles published during five years (2008–2012) in ten pharmacy journals, demonstrating that 52.4% had been indexed without any pharmacy-specific MeSH and 23.6% used the broader MeSH ‘Pharmacists’, which was insufficient to ascertain the objective of the study.^12^ Several reasons associated to NLM cataloging practices could be in the origin this poor MeSH assignment in pharmacy practice articles, but the use of inconsistent terminology in this field^13^ was mentioned as a barrier whether to claim for new MeSH terms or for a more accurate MeSH assignment.^11^^,^^14^

In this context, considering that inaccuracies in the indexing of articles can lead to important bias during literature retrieval,^9^ the objective of this study was to further evaluate the use of ‘Pharmaceutical Services’ MeSH terms in studies assessing the effect of pharmacists' interventions.

## 2 Methods

### 2.1 Study design

This is a meta-research study^15^ aiming at systematically assessing the use of MeSH terms in studies on pharmaceutical services.

#### 2.1.1 Data gathering

The selection of the studies was performed in a multi-step process.

In a first step, the systematic review performed by Bonetti et al.^16^^,^^17^ was updated aiming at identifying all available meta-analyses assessing the impact of pharmaceutical services on economic, humanistic, health outcomes or process indicators. To ensure the consistency, the original research team was involved into the updating process. Systematic searches were conducted in the PubMed, Scopus, and Web of Science (January 2022) without time nor language restrictions (see complete search strategy in Supplemental Material S1). Manual searches in the reference lists of the included studies were also performed. Meta-analyses of interventional or observational primary studies that compared a service provided by pharmacists vs. any health professional or usual care were included. Articles written in non-Roman characters, systematic reviews without meta-analysis, outdated meta-analysis (i.e., only the most recent version was included to avoid duplication results) or studies assessing the effect of interventions provided by a multidisciplinary team without differentiating the role of the pharmacist were excluded. Meta-analysis eligibility process was performed independently by two researchers of the team that conducted the first version. A consensus meeting among these two researchers existed to discuss discrepancies and reach a consensus. If discrepancies persisted, a third researcher of the team decided after a three-party meeting. The final list of meta-analyses was exported into an Excel (Microsoft, Redmond, WA) data sheet.

In a second step, all the primary studies included in the systematic reviews with meta-analyses were identified from the full text version of the meta-analysis article (including online supplementary materials) by a single researcher and compiled in a second Excel data sheet. After removing the duplicates, only primary studies available in PubMed (i.e., with an attributed PMID) were selected for analyses.

#### 2.1.2 Data processing

In a third step, all the MeSH terms allocated to the selected primary studies were systematically extracted from PubMed (date of extraction: 07/03/2022) using the ‘save into PubMed format feature, to create a txt file further imported into a third Excel data sheet. Then, it was assessed whether the MeSH term ‘Pharmaceutical Services' or any of its descendant terms (identified from the NLM controlled vocabulary thesaurus tree - https://www.ncbi.nlm.nih.gov/mesh) had been assigned to the MEDLINE record of each primary study. The complete definition and year of introduction of these terms are depicted in Supplementary material S2.

Additionally, the assignment of other 26 pharmacy-specific MeSH terms previously described in the literature^12^^,^^18^ and potentially related to pharmaceutical services was evaluated (see terms and definitions in Supplementary material S3). It was also identified which of these terms were assigned as a ‘Major MeSH term’ in each article (i.e, they denote the focus of an article and are marked with an asterisk (*); in a search session they can be used to limit results). All the above-mentioned steps were performed in Excel (Microsoft, Redmond, WA) and EndNote (Clarivate, Philadelphia, PA).

#### 2.1.3 Data analysis

Descriptive statistical analyses to characterize the sample of included articles and their assigned MeSH terms (i.e., ‘Pharmaceutical Services’ branch, its descendants or other pharmacy-specific MeSH terms) were performed. Categorical variables were reported as absolute and relative frequencies, and continuous variables with non-normal distribution as the median and interquartile range (IQR). Using a Spearman test for non-parametric correlations, the number of MeSH terms, the number of Major MeSH, and the percentage of Major MeSH with the year of publication were explored.

To assess the potential influence of less pharmacy-centered articles (i.e., without an especial focus on the pharmacist, such as studies evaluating the effect of multidisciplinary teams), a sensitivity analysis (more conservative scenario) was performed. In this case, analyses including only primary articles from meta-analyses that specifically mentioned the word ‘pharmacist’ or ‘pharmacy’ in their declared objectives were re-run. Analyses were conducted in IBM SPSS Statistics v. 24.0 (IBM Corp, Armonk, NY) and *p*-values below 5% were considered statistically significant.

Finally, a social network analysis was conducted to evaluate the co-occurrence of the selected MeSH terms in the included articles using the ForceAtlas2 model of the Gephi (https://gephi.org). ForceAtlas2 is a force directed layout, which simulates a physical system to spatialize a network.^19^ Two networks were created, one to depict the co-occurrence of all the MeSH assigned to the articles, and another to depict co-occurrence between MeSH qualified as Major MeSH.

## 3 Results

**Fig. 1:**
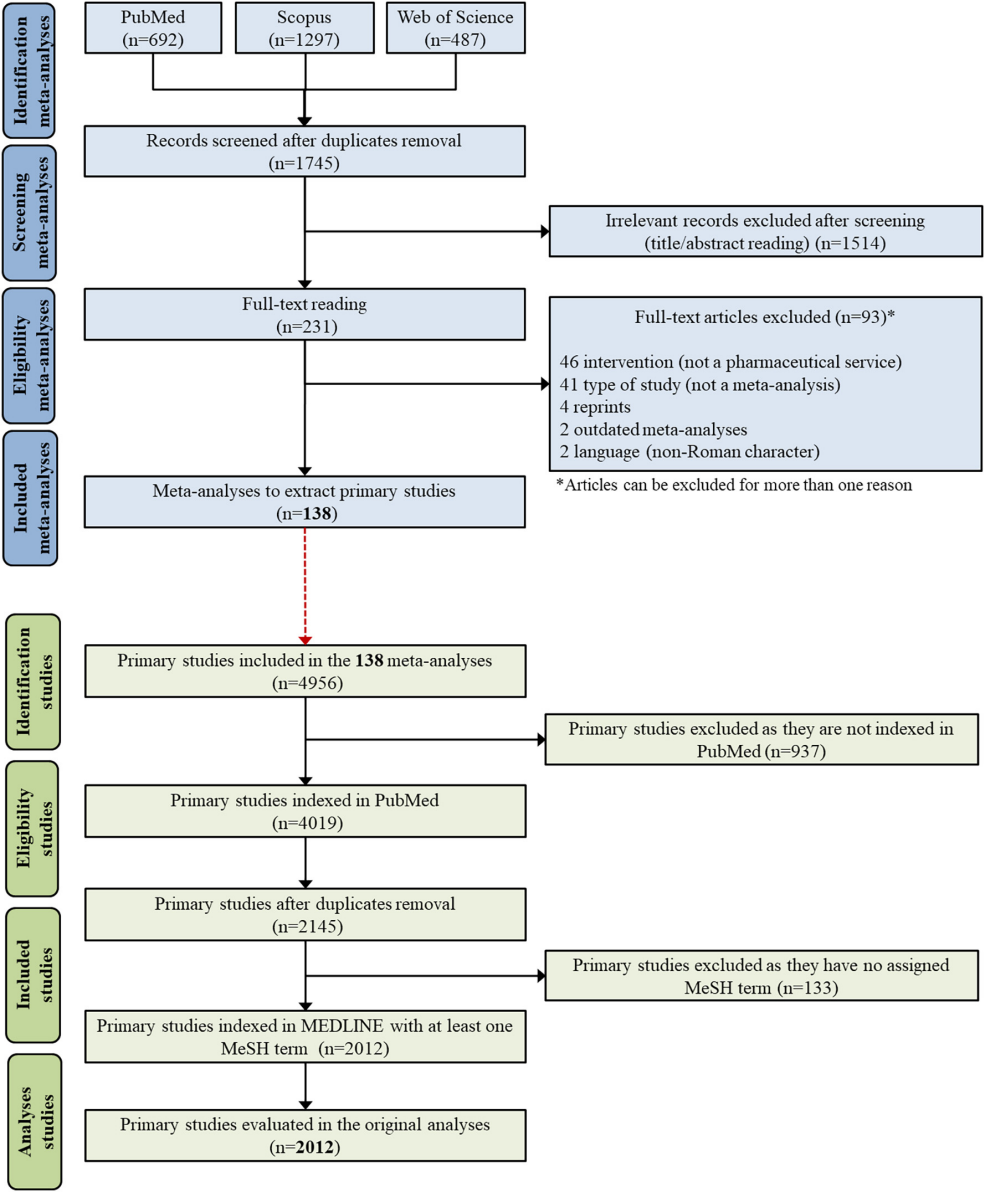
PRISMA Flowchart of the systematic review eligibility process.

After searching for the meta-analyses, a total of 1745 records were retrieved from PubMed, Scopus, and Web of Science after duplicates removal. During the screening process, 1514 records were considered irrelevant. Full-text analysis led to the exclusion of 93 articles, resulting in a total of 138 meta-analyses selected for data extraction (Fig. 1). The complete list of included meta-analyses is available in Supplemental material S4. From these 138 meta-analyses, 4956 primary studies were identified of which 4019 (81.1%) were indexed in PubMed. After removing duplicates, 2145 different primary studies were obtained, of which 2012 (93.8%) had been indexed in MEDLINE with at least one MeSH term assigned.

The 2012 articles were published in 501 different journals with 251 journals publishing only one article, resulting in a typical Bradford's distribution,^20^ which means that a small number of journals (the core or nucleus of the distribution) represents a great proportion of citations. The core section of that distribution contained only about 15 journals comprising 30% of articles (see graphs in Supplemental Material S5). The journals publishing the highest number of articles were Am J Health Syst Pharm (4.6%), Pharmacotherapy (3.4%) and Ann Pharmacother (3.3%) (see the list of the top journals in Supplemental Material S6). The median year of publication of the articles was 2009 (IQR 2003–2013).

Overall, 1893 different MeSH terms were extracted (median number of 15 [IQR 12–18] assigned MeSH terms per article), with 711 (37.6%) of them appearing in only one article (see Supplemental Material S7). A slight positive time trend regarding the number of MeSH assigned per article was observed (Spearman rho = 0.193; *p* < 0.001). Among these terms, 548 different MeSH terms had been classified as ‘Major MeSH term’ (median of 1 [IQR 1–2] Major MeSH term per article), with 267 (48.7%) of them appearing in only one article, with no time trend was observed (Spearman *p* = 0.251). Percentage of MeSH classified as Major MeSH presented a slightly negative trend (Spearman rho = −0.088; p < 0.001) (see Supplemental Material S8-S10).

Results from the sensitivity analyses were similar to those from the overall assessment. In this more conservative scenario, 31 out of 138 meta-analyses were excluded from analyses as they did not present the word ‘pharmacy’ or ‘pharmacist’ in their objective (see Supplemental Material S11). The remaining 107 meta-analyses included 1099 different primary studies. These studies were indexed with 1312 different MeSH terms (520 [39.3%] of them were available in only one article); 354 terms were classified as ‘Major MeSH’ (186 [52.5%] of them appeared in only one article). Table 1 depicts the most frequently assigned MeSH and Major MeSH terms in both original (*n* = 2012 articles) and sensitivity analyses (*n* = 1099 articles).

**Table 1:** Top terms most frequently assigned to the primary studies – overall and sensitivity analyses.

MeSH term	Overall analysis⁎ (*n* = 2012)		Sensitivity analysis⁎⁎ (*n* = 1099)	
	N	%	N	%
Humans	2001	99.45	1089	99.09
Female	1611	80.07	805	73.25
Male	1594	79.22	805	73.25
Middle Aged	1229	61.08	621	56.51
Aged	1093	54.32	589	53.59
Adult	775	38.52	356	32.39
Pharmacists	560	27.83	524	47.68
Patient Education as Topic	477	23.71	237	21.57
Patient Compliance	437	21.72	131	11.92
Treatment Outcome	414	20.58	191	17.38
Aged, 80 and over	398	19.78	252	22.93
Prospective Studies	326	16.20	209	19.02
Follow-Up Studies	311	15.46	141	12.83
Medication Adherence	302	15.01	–	–
Hypertension	264	13.12	–	–
Pharmacy Service, Hospital	232	11.53	215	19.56
Quality of Life	206	10.24	110	10.01
Community Pharmacy Services	205	10.19	169	15.29
Adolescent	203	10.09	–	–
Surveys and Questionnaires	191	9.49	–	–


Major MeSH term	Overall analysis⁎ (n = 2012)		Sensitivity analysis⁎⁎ (n = 1099)	
	N	%	N	%
Pharmacists	297	14.76	283	79.94
Patient Compliance	203	10.09	43	12.15
Patient Education as Topic	153	7.60	69	19.49
Medication Adherence	127	6.31	42	10.73
Community Pharmacy Services	68	3.38	67	18.93
Professional Role	63	3.13	59	16.67
Pharmacy Service, Hospital	53	2.63	50	14.12
Pharmaceutical Services	50	2.49	47	13.28
Patient Care Team	49	2.44	42	11.86
Quality of Life	46	2.29	28	7.91
Patient Discharge	45	2.24	32	9.04
Disease Management	41	2.04	18	5.08
Self Care	40	1.99	–	–
Counseling	39	1.94	–	–
Outcome Assessment, Health Care	38	1.89	23	6.50
Drug-Related Side Effects and Adverse Reactions	33	1.64	28	7.91
Health Knowledge, Attitudes, Practice	33	1.64	–	–
Polypharmacy	32	1.59	18	5.08
Telephone	31	1.54	–	–
Drug Prescriptions	27	1.34	18	5.08
Physicians	–	–	25	7.06
Pharmacies	–	–	20	5.65
Drug Utilization Review	–	–	20	5.65
Drug Utilization	–	–	20	5.65
Drug Prescriptions	–	–	18	5.08

– terms not included in the top terms of each analysis.

⁎ Terms assigned to the 2012 articles included in the 138 meta-analyses.

⁎⁎ Terms assigned to the 1099 articles included in the 107 meta-analyses presenting the words ‘pharmacist’ or ‘pharmacy’ in the objective.

Only 36.6% (*n* = 736/2012) and 58.1% (*n* = 338/1099) of studies were indexed with one MeSH term from the ‘Pharmaceutical Services’ branch of the hierarchic tree in the overall and sensitivity analyses, respectively. In 231 (11.5%) and 199 (18.1%) cases, these MeSH were classified as a ‘Major MeSH’ term, respectively. The most frequently used MeSH terms – yet with frequencies lower than 15%, were ‘Pharmacy Service, Hospital’, ‘Community Pharmacy Services’, and ‘Pharmaceutical Services’, appearing in around 11%, 10% and 9% of articles in the original analyses, respectively. These figures were slightly higher in the sensitivity analyses, with values of around 20%, 18%, 15%, respectively as showed in Table 2. No study with the terms ‘Prescription Drug Monitoring Programs’ and ‘Pharmaceutical Services, Online’ were found.

**Table 2:** Frequency of ‘Pharmaceutical Service’ terms branch assigned to primary studies – overall and sensitivity analyses.

MeSH term	Overall analysis⁎ (*n* = 2012)		Sensitivity analysis⁎⁎ (*n* = 1099)	
	N	%	N	%
Pharmaceutical Services	181	9.00	168	15.29
Community Pharmacy Services	205	10.19	192	17.47
Drug Information Services	9	0.45	8	0.73
Adverse Drug Reaction Reporting Systems	11	0.55	7	0.64
Clinical Pharmacy Information Systems	18	0.89	13	1.18
Prescription Drug Monitoring Programs	0	0	0	0
Medication Therapy Management	68	3.38	46	4.19
Pharmaceutical Services, Online	0	0	0	0
Pharmacy Service, Hospital	232	11.53	215	19.56
Prescriptions	5	0.25	4	0.36
Drug Prescriptions	108	5.37	78	7.10
Drug Substitution	4	0.19	2	0.18
Electronic Prescribing	9	0.45	1	0.09


Major MeSH term	Overall analysis⁎ (n = 2012)		Sensitivity analysis⁎⁎ (n = 1099)	
	N	%	N	%
Pharmaceutical Services	50	2.49	47	4.28
Community Pharmacy Services	68	3.38	67	6.10
Drug Information Services	2	0.10	2	0.18
Adverse Drug Reaction Reporting Systems	4	0.20	3	0.27
Clinical Pharmacy Information Systems	9	0.45	5	0.45
Prescription Drug Monitoring Programs	0	0	0	0
Medication Therapy Management	21	1.04	15	1.36
Pharmaceutical Services, Online	0	0	0	0
Pharmacy Service, Hospital	53	2.63	50	4.55
Prescriptions	1	0.05	0	0
Drug Prescriptions	1	0.05	18	1.64
Drug Substitution	27	1.34	1	0.09
Electronic Prescribing	1	0.05	0	0

⁎ Terms assigned to the 2012 articles included in the 138 meta-analyses.

⁎⁎ Terms assigned to the 1099 articles included in the 107 meta-analyses presenting the words ‘pharmacist’ or ‘pharmacy’ in the objective.

Among other pharmacy-specific MeSH terms not included in the ‘Pharmaceutical Services’ branch’, ‘Pharmacists' was the most frequent MeSH available in 27.8% and around 48% of articles in the original and sensitivity analyses, respectively, while ‘Pharmacies' was the second most used, yet in only 3% and 4.5% of studies, respectively. Other terms such as ‘Insurance, Pharmaceutical Services', ‘Pharmacy’ and ‘Education, Pharmacy’ were poorly assigned (<1% of articles) (see Table 3).

**Table 3:** Frequency of pharmacy-specific MeSH terms assigned to primary studies – overall and sensitivity analyses.

MeSH term	Overall analysis⁎ (n = 2012)		Sensitivity analysis⁎⁎ (*n* = 1099)	
	N	%	N	%
Behind-the-Counter Drugs	0	0.0	0	0
Dictionaries, Pharmaceutic	0	0.0	0	0
Drug Compounding	1	0.0	0	0
Education, Pharmacy	8	0.4	6	0.55
Education, Pharmacy, Continuing	4	0.2	4	0.36
Education, Pharmacy, Graduate	0	0.0	0	0
Ethics, Pharmacy	0	0.0	0	0
Evidence-Based Pharmacy Practice	0	0.0	0	0
Faculty, Pharmacy	0	0.0	0	0
Fees, Pharmaceutical	1	0.0	1	0.09
History of Pharmacy	0	0.0	0	0
Insurance, Pharmaceutical Services	11	0.5	6	0.55
Legislation, Pharmacy	4	0.2	4	0.36
Licensure, Pharmacy	0	0.0	0	0
Pharmacies	55	2.7	48	4.37
Pharmacists	560	27.8	524	47.68
Pharmacists' Aides	0	0.0	0	0
Pharmacy	7	0.3	5	0.45
Pharmacy Administration	2	0.1	2	0.18
Pharmacy Research	0	0.0	0	0
Pharmacy and Therapeutics Committee	2	0.1	2	0.18
Practice Patterns, Pharmacists’	0	0.0	0	0
Schools, Pharmacy	1	0.0	1	0.09
Societies, Pharmaceutical	0	0.0	0	0
Students, Pharmacy	2	0.1	2	0.18
Technology, Pharmaceutical	0	0.0	0	0

⁎ Terms assigned to the 2012 articles included in the 138 meta-analyses.

⁎⁎ Terms assigned to the 1099 articles included in the 107 meta-analyses presenting the words ‘pharmacist’ or ‘pharmacy’ in the objective.

**Fig. 2:**
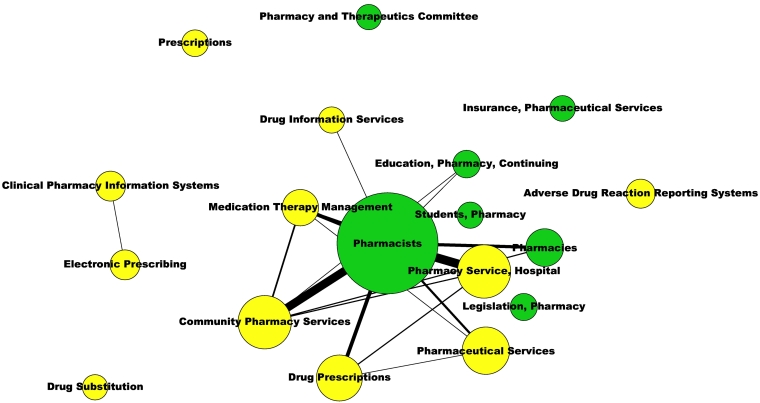
Co-occurrence network of the pharmacy-related MeSH terms assigned to the 2012 articles included in the 138 meta-analyses. Yellow nodes represent the MeSH terms of the ‘Pharmaceutical Services’ branch. Green nodes represent pharmacy-specific MeSH terms.

**Fig. 3:**
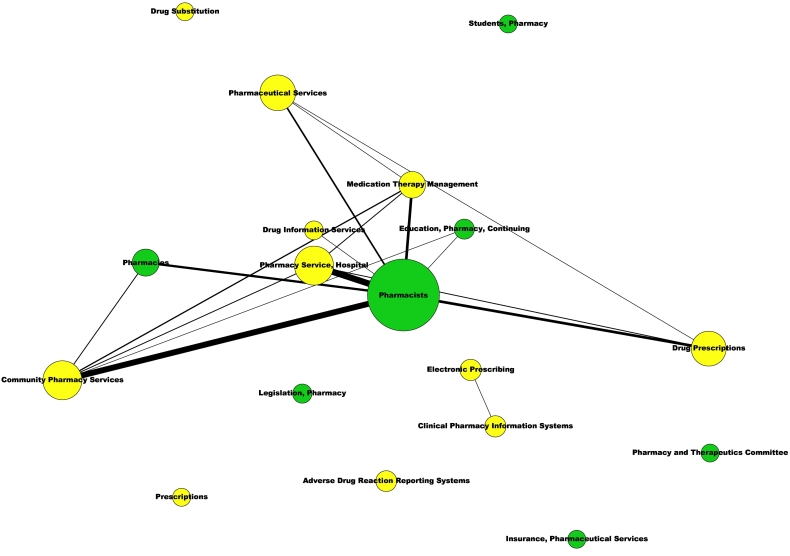
Co-occurrence network of the pharmacy-related MeSH terms classified as Major MeSH assigned to the 2012 articles included in the 138 meta-analyses. Yellow nodes represent the MeSH terms of the ‘Pharmaceutical Services’ branch. Green nodes represent pharmacy-specific MeSH terms.

Social network analyses provided two networks including all the MeSH (Supplemental Material S12) and all the MeSH classified as ‘Major MeSH’ (Supplemental Material S13) that demonstrate a weak association between pharmacy-specific MeSH and ‘Pharmaceutical services’ branch MeSH. This weak association is more evident in the networks including only these two groups of MeSH terms (Fig. 2, Fig. 3), where became evident that only the MeSH ‘Pharmacists’ was slightly linked to some of the ‘Pharmaceutical services’ MeSH terms (*n* = 6).

## 4 Discussion

This meta-research analysis of MEDLINE records corresponding to the over 2000 primary studies included in all the meta-analyses evaluating the impact of pharmacists' interventions demonstrated a poor use of the MeSH ‘Pharmaceutical services’ and its descendant terms. The concomitant scarce assignment of other pharmacy-specific MeSH, together with the weak co-occurrence of these terms may challenge the retrieval of pharmacists' intervention articles.

The main goal of a bibliographic database (e.g., MEDLINE) should be to facilitate the visibility and retrieval of scientific literature. To accomplish this goal, having a robust search engine, like PubMed, may not be sufficient. Controlled vocabularies of subject headings were created to standardize terms to index and catalog scientific articles and thus avoid the terminology variations among researchers. NLM MeSH thesaurus is a perfect example of well-organized subject headings controlled vocabulary. MeSH thesaurus hierarchic organization allows the ‘automatic explosion’ feature, which means that, unless specifically blocked by the user, “PubMed automatically searches the MeSH headings as well as the more specific terms beneath that heading in the MeSH hierarchy”.^21^

Although previous studies criticized the coverage of pharmacy practice area by MeSH thesaurus, several pharmacy-specific MeSH exist.^9^ As stated by the MeSH staff, to create a new MeSH term, a consistent use of that term in literature should be evidenced.^11^ Therefore, the use of non-standardized terminology, frequently *ad hoc* created by a research group to defend their innovation, may be weakening the request for new pharmacy-related MeSH terms. Among the reasons provided by MeSH staff to not creating new pharmacy-specific MeSH was the potential use of the immediately ascendant MeSH (‘father MeSH’) together with the MeSH term ‘Pharmacists’. To ensure the assignment of that ascendent MeSH to pharmacy articles, MeSH staff created an artificial artifact consisting in adding the suggested pharmacy-specific MeSH as a concept to the ascendant MeSH. For example, the 2014 analysis suggested the creation of ‘Pharmacist-Patient Relations’ as descendant of ‘Professional-Patient Relations’, similarly to what happens with ‘Nurse-Patient Relations’ and ‘Dentist-Patient Relations’.^9^ After this suggestion, MeSH staff preferred adding ‘Pharmacist-Patient Relation’ as a ‘Broader Concept’ (strictly a synonym, as defined by NLM)^22^ of the MeSH ‘Professional-Patient Relations’, which does not happen with any other healthcare profession. Thus, automatic indexing systems will assign the ascendant MeSH to any article about pharmacist-patient relationships.

A group of the existing pharmacy-specific MeSH are organized in a branch of the hierarchic tree under the MeSH ‘Pharmaceutical services’. One could expect that all the articles covering pharmacists' interventions would be indexed with the ‘Pharmaceutical services’ MeSH term or one of its more specific descendants. However, the present study demonstrated that this is far from being the routine, with only one in three articles in the overall analysis being indexed with one of these MeSH terms, which increased to only half of them in the ultra-specific sensitivity analysis. Moreover, other available terms in the MeSH thesaurus, including ‘Pharmacy’ and ‘Education, Pharmacy’ were assigned to <3% of articles and had a weak association with the ‘Pharmaceutical services’ branch MeSH, confirming that inaccuracies in tree structure, lack of standard terminology in the field as well as poor allocation practices still exist. While more than two decades ago, MeSH terms were exclusively assigned by humans, in 2002 NLM introduced the Medical Text Indexer (MTI), a system that uses natural language processing technology to provide recommendations to NLM indexers.^23^ This project is being increasingly implemented resulting in the Medical Text Indexer First Line Indexing (MTIFL),^24^ a fully automated system that will no longer use humans after mid-2022.^25^ A myriad of systems were designed to identify the most appropriate MeSH terms to articles, some using as source the full-text articles, other using specifically created summaries.^26^, ^27^, ^28^ However, NLM MTIFL uses only articles' titles and the abstracts, both written by the authors. Therefore, the use of non-standardized terms, is not only weakening the request for new MeSH terms, but also the accurate article indexing.

Probably, the most feasible solution to the poor indexing in pharmacy practice area would require pharmacy practice researchers' global collaboration aiming to overcome several weaknesses of pharmacy practice literature. The use of standardized terminology will be crucial to improve indexing. A potential root cause of the inconsistent terminology in pharmacy practice could be the huge journal scattering in the area. As in previous studies,^12^^,^^29^ articles included in the present study were published in >500 different journals. Authors referred that publishing in non-pharmacy journals will increase the visibility of their work by other professionals. The ‘augmenting the visibility by publishing elsewhere’ misconception not only ignores that bibliographic databases like MEDLINE are the way articles are commonly identified, but also impedes the potential improvement that colleague editors and reviewers could provide to the original manuscript during the editorial process.

### 4.1 Limitations

The results obtained in this study represent only the literature included in meta-analyses of pharmacists' interventions, but there is no reason to think that other pharmacists' intervention articles would perform differently. As in any systematic search, articles may have been not included due to different aspects like those revealed in this article. However, since meta-analyses retrieved with the systematic search were used to obtain a sample of >4000 articles about pharmaceutical services, the potential limitations of the systematic search should have no impact on the conclusions of the study.

## 5 Conclusion

An important proportion of articles reporting pharmacists' intervention studies are not indexed in MEDLINE with any of the MeSH terms from the ‘Pharmaceutical services’ branch of the MeSH thesaurus. Pharmacy practice researchers, editors, and peer reviewers should commit in using and promoting the use of standardized terminology, especially in the new automatic indexing scenario.

## Funding

This research did not receive any specific grant from funding agencies in the public, commercial, or not-for-profit sectors.

## CRediT authorship contribution statement

**Fernanda S. Tonin:** Conceptualization, Methodology, Writing – original draft, Writing – review & editing. **Vanessa Gmünder:** Data curation, Formal analysis, Writing – review & editing. **Aline F. Bonetti:** Data curation, Writing – review & editing. **Antonio M. Mendes:** Validation, Writing – review & editing. **Fernando Fernandez-Llimos:** Conceptualization, Formal analysis, Supervision, Writing – original draft, Writing – review & editing.

## Declaration of Competing Interest

None.

## Appendix A Supplementary data

Supplementary data of the ellegibility process. Image 1
