# Supplementary material for: Use of ‘Pharmaceutical services’ Medical Subject Headings (MeSH) in articles assessing pharmacists' interventions
Source: Explor Res Clin Soc Pharm. 2022 Aug 20;7:100172. doi: 10.1016/j.rcsop.2022.100172 (PMC9445408; doi:10.1016/j.rcsop.2022.100172)
Supplement: Supplementary file 1 — Supplementary data of the ellegibility process. [file mmc1.docx]

**Supplemental Material**

**S1. Search strategies for PubMed, Scopus and Web of Science to retrieve meta-analyses reporting the effect of pharmaceutical services**

| PubMed | |
| --- | --- |
|  | (“pharmaceutical care”[TIAB] OR “pharmacy services”[TIAB] OR “pharmacy service”[TIAB] OR "pharmaceutical services"[MH] OR pharmacists[MH] OR pharmacist*[TIAB]) AND (Meta-analysis[TIAB] OR meta-analyses[TIAB]) |
| Scopus | |
|  | ( TITLE-ABS-KEY ( pharmacist OR "pharmacy services" OR "pharmaceutical services" OR "pharmaceutical care" ) ) AND ( TITLE-ABS-KEY ( meta-analysis OR meta-analyses ) ) |
| Web of Science | |
|  | (Meta-analysis) AND (pharmacists OR "pharmaceutical service" OR "pharmaceutical care") |

**S2. Overall hierarchy and definitions of the MeSH term Pharmaceutical Services and descendent terms and year of introduction in MEDLINE**


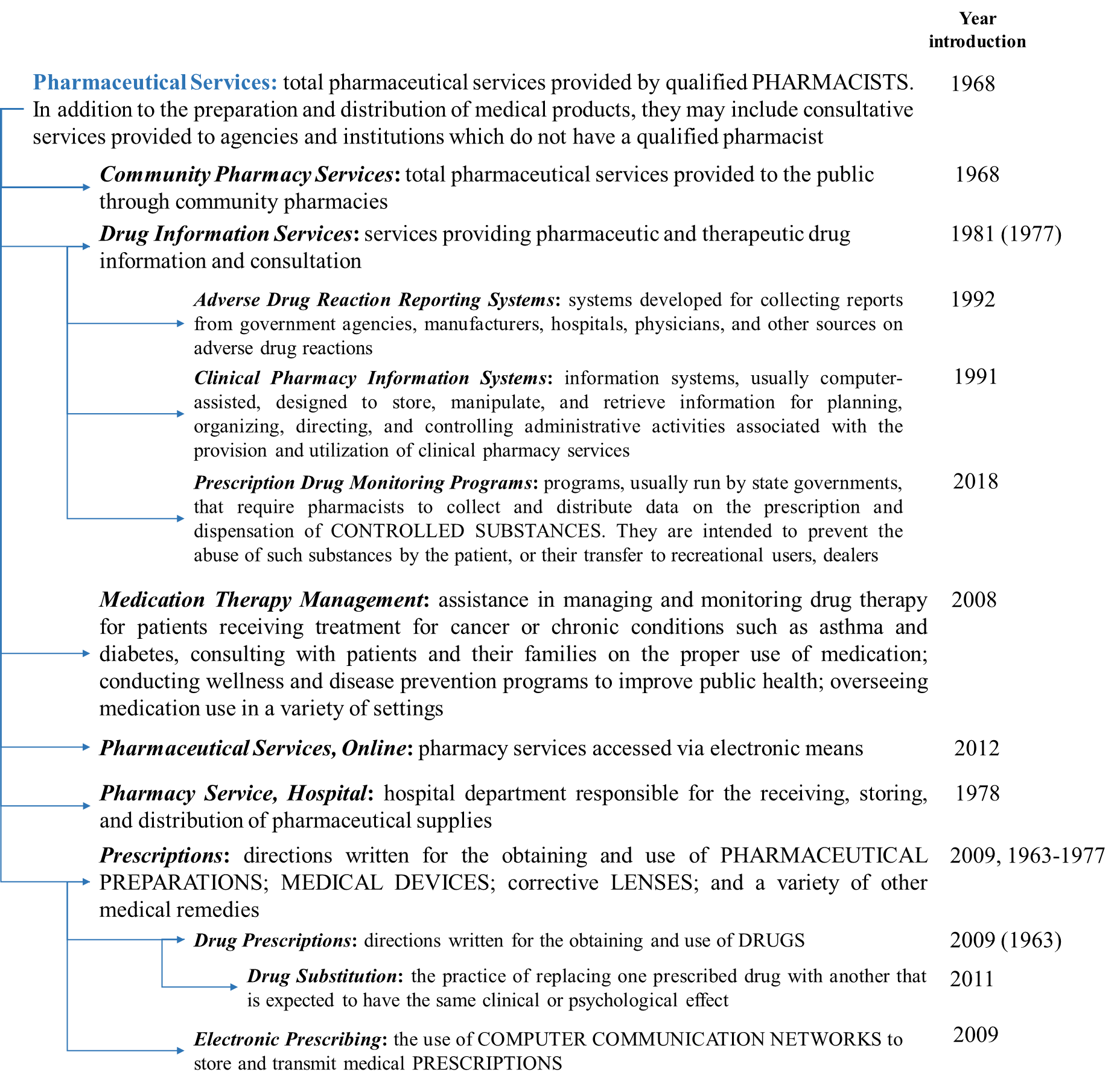


**S3. Pharmacy-specific MeSH terms and year of introduction in MEDLINE**


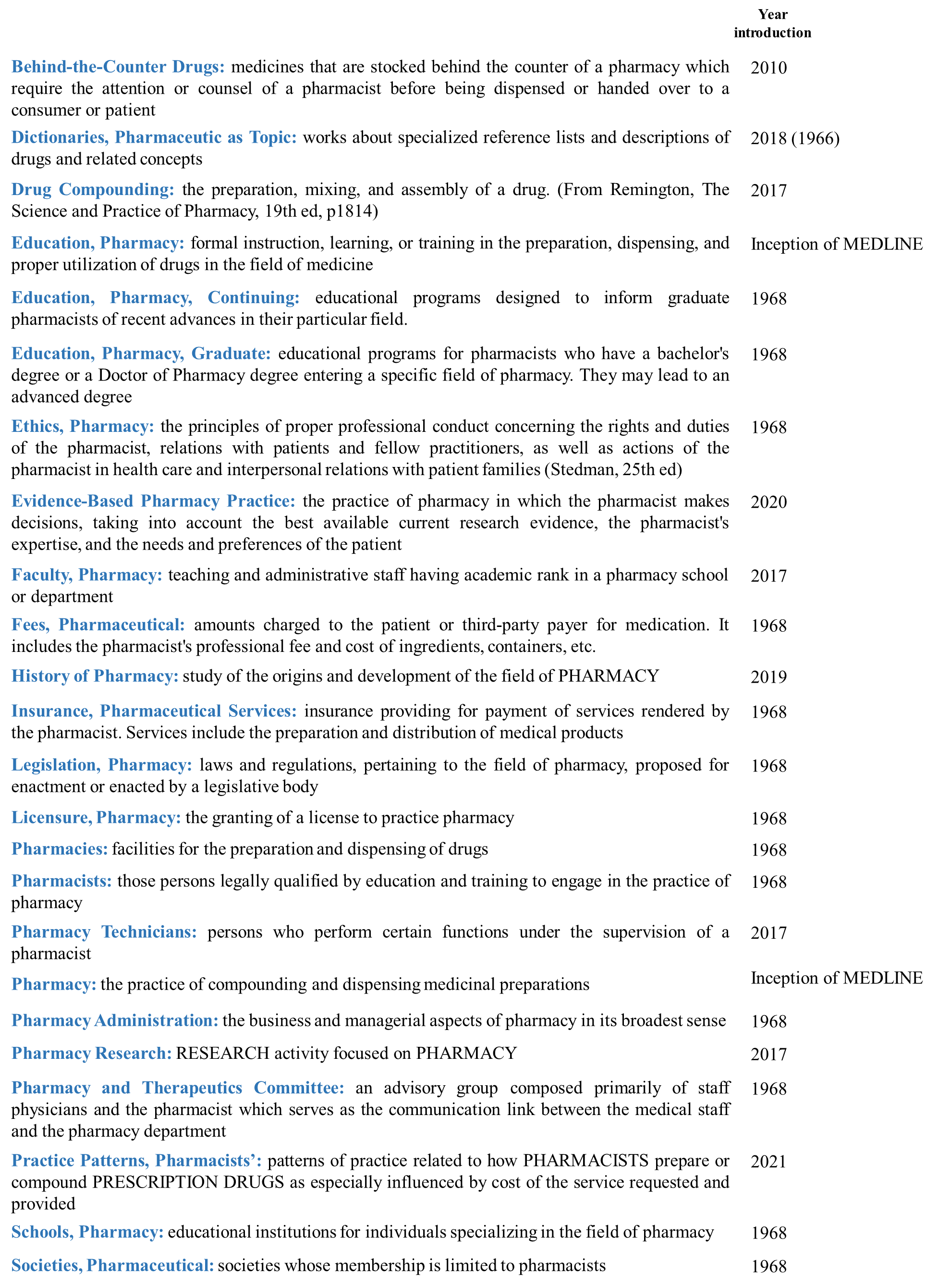


**S4. List of the 138 meta-analyses included for analysis**

| **Id** | **PMID** | **Article** |
| --- | --- | --- |
| 1 | 30538430 | Bukhsh A, Tan XY, Chan KG, Lee LH, Goh BH, Khan TM. Effectiveness of pharmacist-led educational interventions on self-care activities and glycemic control of type 2 diabetes patients: a systematic review and meta-analysis. Patient Prefer Adherence. 2018;12():2457-74 |
| 2 | 30515250 | Ramsubeik K, Ramrattan LA, Kaeley GS, Singh JA. Effectiveness of healthcare educational and behavioral interventions to improve gout outcomes: a systematic review and meta-analysis. Ther Adv Musculoskelet Dis. 2018;10(12):235-52 |
| 3 | 30389320 | Dawoud DM, Smyth M, Ashe J, Strong T, Wonderling D, Hill J, Varia M, Dyer P, Bion J. Effectiveness and cost effectiveness of pharmacist input at the ward level: a systematic review and meta-analysis. Res Social Adm Pharm. 2018;(): |
| 4 | 30362920 | Dokbua S, Dilokthornsakul P, Chaiyakunapruk N, Saini B, Krass I, Dhippayom T. Effects of an Asthma Self-Management Support Service Provided by Community Pharmacists: A Systematic Review and Meta-Analysis. J Manag Care Spec Pharm. 2018;24(11):1184-96 |
| 5 | 30319263 | Jeong S, Lee M, Ji E. Effect of pharmaceutical care interventions on glycemic control in patients with diabetes: a systematic review and meta-analysis. Ther Clin Risk Manag. 2018;14():1813-29 |
| 9 | 30178872 | de Barra M, Scott CL, Scott NW, Johnston M, de Bruin M, Nkansah N, Bond CM, Matheson CI, Rackow P, Williams AJ, Watson MC. Pharmacist services for non-hospitalised patients. Cochrane Database Syst Rev. 2018;9():CD013102 |
| 13 | 29976652 | Mes MA, Katzer CB, Chan AHY, Wileman V, Taylor SJC, Horne R. Pharmacists and medication adherence in asthma: a systematic review and meta-analysis. Eur Respir J. 2018;52(2): |
| 21 | 29692730 | Bukhsh A, Khan TM, Lee SWH, Lee LH, Chan KG, Goh BH. Efficacy of Pharmacist Based Diabetes Educational Interventions on Clinical Outcomes of Adults With Type 2 Diabetes Mellitus: A Network Meta-Analysis. Front Pharmacol. 2018;9():339 |
| 37 | 29326185 | Nguyen T, Nguyen HQ, Widyakusuma NN, Nguyen TH, Pham TT, Taxis K. Enhancing prescribing of guideline-recommended medications for ischaemic heart diseases: a systematic review and meta-analysis of interventions targeted at healthcare professionals. BMJ Open. 2018;8(1):e018271 |
| 38 | 29311916 | van Eikenhorst L, Taxis K, van Dijk L, de Gier H. Pharmacist-Led Self-management Interventions to Improve Diabetes Outcomes. A Systematic Literature Review and Meta-Analysis. Front Pharmacol. 2017;8():891 |
| 39 | 29265170 | Gray SL, Hart LA, Perera S, Semla TP, Schmader KE, Hanlon JT. Meta-analysis of Interventions to Reduce Adverse Drug Reactions in Older Adults. J Am Geriatr Soc. 2018;66(2):282-8 |
| 40 | 29248878 | McNab D, Bowie P, Ross A, MacWalter G, Ryan M, Morrison J. Systematic review and meta-analysis of the effectiveness of pharmacist-led medication reconciliation in the community after hospital discharge. BMJ Qual Saf. 2018;27(4):308-20 |
| 42 | 29224636 | Yaghoubi M, Mansell K, Vatanparastc H, Steeves M, Zeng W, Farag M. Effects of Pharmacy-Based Interventions on the Control and Management of Diabetes in Adults: A Systematic Review and Meta-Analysis. Can J Diabetes. 2017;41(6):628-41 |
| 43 | 29145935 | Hou K, Yang H, Ye Z, Wang Y, Liu L, Cui X. Effectiveness of Pharmacist-led Anticoagulation Management on Clinical Outcomes: A Systematic Review and Meta-Analysis. J Pharm Pharm Sci. 2017;20(1):378-96 |
| 48 | 28948839 | Deters MA, Laven A, Castejon A, Doucette WR, Ev LS, Krass I, Mehuys E, Obarcanin E, Schwender H, Laeer S. Effective Interventions for Diabetes Patients by Community Pharmacists: A Meta-analysis of Pharmaceutical Care Components. Ann Pharmacother. 2018;52(2):198-211 |
| 53 | 28851770 | Mbeye NM, Adetokunboh O, Negussie E, Kredo T, Wiysonge CS. Shifting tasks from pharmacy to non-pharmacy personnel for providing antiretroviral therapy to people living with HIV: a systematic review and meta-analysis. BMJ Open. 2017;7(8):e015072 |
| 56 | 28671909 | De Oliveira GS, Jr., Castro-Alves LJ, Kendall MC, McCarthy R. Effectiveness of Pharmacist Intervention to Reduce Medication Errors and Health-Care Resources Utilization After Transitions of Care: A Meta-analysis of Randomized Controlled Trials. J Patient Saf. 2017;(): |
| 58 | 28622997 | Readdean KC, Heuer AJ, Scott Parrott J. Effect of pharmacist intervention on improving antidepressant medication adherence and depression symptomology: A systematic review and meta-analysis. Res Social Adm Pharm. 2018;14(4):321-31 |
| 61 | 28599601 | Rodrigues CR, Harrington AR, Murdock N, Holmes JT, Borzadek EZ, Calabro K, Martin J, Slack MK. Effect of Pharmacy-Supported Transition-of-Care Interventions on 30-Day Readmissions: A Systematic Review and Meta-analysis. Ann Pharmacother. 2017;51(10):866-89 |
| 63 | 28573873 | Fazel MT, Bagalagel A, Lee JK, Martin JR, Slack MK. Impact of Diabetes Care by Pharmacists as Part of Health Care Team in Ambulatory Settings: A Systematic Review and Meta-analysis. Ann Pharmacother. 2017;51(10):890-907 |
| 70 | 28315760 | Conn VS, Ruppar TM. Medication adherence outcomes of 771 intervention trials: Systematic review and meta-analysis. Prev Med. 2017;99():269-76 |
| 75 | 28233442 | Van Spall HGC, Rahman T, Mytton O, Ramasundarahettige C, Ibrahim Q, Kabali C, Coppens M, Brian Haynes R, Connolly S. Comparative effectiveness of transitional care services in patients discharged from the hospital with heart failure: a systematic review and network meta-analysis. Eur J Heart Fail. 2017;19(11):1427-43 |
| 80 | 28095780 | Huiskes VJ, Burger DM, van den Ende CH, van den Bemt BJ. Effectiveness of medication review: a systematic review and meta-analysis of randomized controlled trials. BMC Fam Pract. 2017;18(1):5 |
| 84 | 28000212 | van Driel ML, Morledge MD, Ulep R, Shaffer JP, Davies P, Deichmann R. Interventions to improve adherence to lipid-lowering medication. Cochrane Database Syst Rev. 2016;12():CD004371 |
| 89 | 27873322 | Weeks G, George J, Maclure K, Stewart D. Non-medical prescribing versus medical prescribing for acute and chronic disease management in primary and secondary care. Cochrane Database Syst Rev. 2016;11():CD011227 |
| 95 | 27696540 | Loh ZW, Cheen MH, Wee HL. Humanistic and economic outcomes of pharmacist-provided medication review in the community-dwelling elderly: A systematic review and meta-analysis. J Clin Pharm Ther. 2016;41(6):621-33 |
| 96 | 27677651 | Zhou S, Sheng XY, Xiang Q, Wang ZN, Zhou Y, Cui YM. Comparing the effectiveness of pharmacist-managed warfarin anticoagulation with other models: a systematic review and meta-analysis. J Clin Pharm Ther. 2016;41(6):602-11 |
| 97 | 27765379 | Isenor JE, Edwards NT, Alia TA, Slayter KL, MacDougall DM, McNeil SA, Bowles SK. Impact of pharmacists as immunizers on vaccination rates: A systematic review and meta-analysis. Vaccine. 2016;34(47):5708-23 |
| 98 | 27566970 | Sawangjit R, Khan TM, Chaiyakunapruk N. Effectiveness of pharmacy-based needle/syringe exchange programme for people who inject drugs: a systematic review and meta-analysis. Addiction. 2017;112(2):236-47 |
| 105 | 27511835 | Renaudin P, Boyer L, Esteve MA, Bertault-Peres P, Auquier P, Honore S. Do pharmacist-led medication reviews in hospitals help reduce hospital readmissions? A systematic review and meta-analysis. Br J Clin Pharmacol. 2016;82(6):1660-73 |
| 108 | 27450138 | Baroy J, Chung D, Frisch R, Apgar D, Slack MK. The impact of pharmacist immunization programs on adult immunization rates: A systematic review and meta-analysis. J Am Pharm Assoc. 2016;56(4):418-26 |
| 109 | 27363846 | Mohammed MA, Moles RJ, Chen TF. Impact of Pharmaceutical Care Interventions on Health-Related Quality-of-Life Outcomes: A Systematic Review and Meta-analysis. Ann Pharmacother. 2016;50(10):862-81 |
| 128 | 26969094 | Conn VS, Ruppar TM, Chase JD. Blood pressure outcomes of medication adherence interventions: systematic review and meta-analysis. J Behav Med. 2016;39(6):1065-75 |
| 131 | 26954666 | Kang JE, Han NY, Oh JM, Jin HK, Kim HA, Son IJ, Rhie SJ. Pharmacist-involved care for patients with heart failure and acute coronary syndrome: a systematic review with qualitative and quantitative meta-analysis. J Clin Pharm Ther. 2016;41(2):145-57 |
| 133 | 26928025 | Brown TJ, Todd A, OMalley C, Moore HJ, Husband AK, Bambra C, Kasim A, Sniehotta FF, Steed L, Smith S, Nield L, Summerbell CD. Community pharmacy-delivered interventions for public health priorities: a systematic review of interventions for alcohol reduction, smoking cessation and weight management, including meta-analysis for smoking cessation. BMJ Open. 2016;6(2):e009828 |
| 135 | 26913812 | Mekonnen AB, McLachlan AJ, Brien JA. Pharmacy-led medication reconciliation programmes at hospital transitions: a systematic review and meta-analysis. J Clin Pharm Ther. 2016;41(2):128-44 |
| 136 | 26908524 | Mekonnen AB, McLachlan AJ, Brien JA. Effectiveness of pharmacist-led medication reconciliation programmes on clinical outcomes at hospital transitions: a systematic review and meta-analysis. BMJ Open. 2016;6(2):e010003 |
| 159 | 26260916 | Wang T, Benedict N, Olsen KM, Luan R, Zhu X, Zhou N, Tang H, Yan Y, Peng Y, Shi L. Effect of critical care pharmacists intervention on medication errors: A systematic review and meta-analysis of observational studies. J Crit Care. 2015;30(5):1101-6 |
| 168 | 25868941 | Meid AD, Lampert A, Burnett A, Seidling HM, Haefeli WE. The impact of pharmaceutical care interventions for medication underuse in older people: a systematic review and meta-analysis. Br J Clin Pharmacol. 2015;80(4):768-76 |
| 170 | 25810127 | Rocha BS, Silveira MP, Moraes CG, Kuchenbecker RS, Dal-Pizzol TS. Pharmaceutical interventions in antiretroviral therapy: systematic review and meta-analysis of randomized clinical trials. J Clin Pharm Ther. 2015;40(3):251-8 |
| 175 | 25581134 | Hohl CM, Wickham ME, Sobolev B, Perry JJ, Sivilotti ML, Garrison S, Lang E, Brasher P, Doyle-Waters MM, Brar B, Rowe BH, Lexchin J, Holland R. The effect of early in-hospital medication review on health outcomes: a systematic review. Br J Clin Pharmacol. 2015;80(1):51-61 |
| 180 | 25401788 | Viswanathan M, Kahwati LC, Golin CE, Blalock SJ, Coker-Schwimmer E, Posey R, Lohr KN. Medication therapy management interventions in outpatient settings: a systematic review and meta-analysis. JAMA Intern Med. 2015;175(1):76-87 |
| 182 | 25330865 | Zhong H, Ni XJ, Cui M, Liu XY. Evaluation of pharmacist care for patients with chronic obstructive pulmonary disease: a systematic review and meta-analysis. Int J Clin Pharm. 2014;36(6):1230-40 |
| 193 | 24966032 | Cheema E, Sutcliffe P, Singer DR. The impact of interventions by pharmacists in community pharmacies on control of hypertension: a systematic review and meta-analysis of randomized controlled trials. Br J Clin Pharmacol. 2014;78(6):1238-47 |
| 198 | 24749899 | Saba M, Diep J, Saini B, Dhippayom T. Meta-analysis of the effectiveness of smoking cessation interventions in community pharmacy. J Clin Pharm Ther. 2014;39(3):240-7 |
| 201 | 24721801 | Santschi V, Chiolero A, Colosimo AL, Platt RW, Taffe P, Burnier M, Burnand B, Paradis G. Improving blood pressure control through pharmacist interventions: a meta-analysis of randomized controlled trials. J Am Heart Assoc. 2014;3(2):e000718 |
| 217 | 24196278 | Thomas R, Huntley AL, Mann M, Huws D, Elwyn G, Paranjothy S, Purdy S. Pharmacist-led interventions to reduce unplanned admissions for older people: a systematic review and meta-analysis of randomised controlled trials. Age Ageing. 2014;43(2):174-87 |
| 218 | 24161491 | Tan EC, Stewart K, Elliott RA, George J. Pharmacist services provided in general practice clinics: a systematic review and meta-analysis. Res Social Adm Pharm. 2014;10(4):608-22 |
| 234 | 23796001 | Lee JK, Slack MK, Martin J, Ehrman C, Chisholm-Burns M. Geriatric patient care by U.S. pharmacists in healthcare teams: systematic review and meta-analyses. J Am Geriatr Soc. 2013;61(7):1119-27 |
| 242 | 23594037 | Hatah E, Braund R, Tordoff J, Duffull SB. A systematic review and meta-analysis of pharmacist-led fee-for-services medication review. Br J Clin Pharmacol. 2014;77(1):102-15 |
| 256 | 23173140 | Santschi V, Chiolero A, Paradis G, Colosimo AL, Burnand B. Pharmacist interventions to improve cardiovascular disease risk factors in diabetes: a systematic review and meta-analysis of randomized controlled trials. Diabetes Care. 2012;35(12):2706-17 |
| 279 | 21911628 | Santschi V, Chiolero A, Burnand B, Colosimo AL, Paradis G. Impact of pharmacist care in the management of cardiovascular disease risk factors: a systematic review and meta-analysis of randomized trials. Arch Intern Med. 2011;171(16):1441-53 |
| 287 | 21610491 | Bennett MI, Bagnall AM, Raine G, Closs SJ, Blenkinsopp A, Dickman A, Ellershaw J. Educational interventions by pharmacists to patients with chronic pain: systematic review and meta-analysis. Clin J Pain. 2011;27(7):623-30 |
| 298 | 21258029 | Morgado MP, Morgado SR, Mendes LC, Pereira LJ, Castelo-Branco M. Pharmacist interventions to enhance blood pressure control and adherence to antihypertensive therapy: Review and meta-analysis. Am J Health Syst Pharm. 2011;68(3):241-53 |
| 299 | 21205952 | Rubio-Valera M, Serrano-Blanco A, Magdalena-Belio J, Fernandez A, Garcia-Campayo J, Pujol MM, del Hoyo YL. Effectiveness of pharmacist care in the improvement of adherence to antidepressants: a systematic review and meta-analysis. Ann Pharmacother. 2011;45(1):39-48 |
| 308 | 20961643 | Collins C, Limone BL, Scholle JM, Coleman CI. Effect of pharmacist intervention on glycemic control in diabetes. Diabetes Res Clin Pract. 2011;92(2):145-52 |
| 309 | 20831620 | Saokaew S, Permsuwan U, Chaiyakunapruk N, Nathisuwan S, Sukonthasarn A. Effectiveness of pharmacist-participated warfarin therapy management: a systematic review and meta-analysis. J Thromb Haemost. 2010;8(11):2418-27 |
| 311 | 20720510 | Chisholm-Burns MA, Kim Lee J, Spivey CA, Slack M, Herrier RN, Hall-Lipsy E, Graff Zivin J, Abraham I, Palmer J, Martin JR, Kramer SS, Wunz T. US pharmacists effect as team members on patient care: systematic review and meta-analyses. Med Care. 2010;48(10):923-33 |
| 326 | 19858431 | Carter BL, Rogers M, Daly J, Zheng S, James PA. The potency of team-based care interventions for hypertension: a meta-analysis. Arch Intern Med. 2009;169(19):1748-55 |
| 341 | 18682540 | Machado M, Nassor N, Bajcar JM, Guzzo GC, Einarson TR. Sensitivity of patient outcomes to pharmacist interventions. Part III: systematic review and meta-analysis in hyperlipidemia management. Ann Pharmacother. 2008;42(9):1195-207 |
| 353 | 18093253 | Holland R, Desborough J, Goodyer L, Hall S, Wright D, Loke YK. Does pharmacist-led medication review help to reduce hospital admissions and deaths in older people? A systematic review and meta-analysis. Br J Clin Pharmacol. 2008;65(3):303-16 |
| 357 | 17925496 | Machado M, Bajcar J, Guzzo GC, Einarson TR. Sensitivity of patient outcomes to pharmacist interventions. Part II: Systematic review and meta-analysis in hypertension management. Ann Pharmacother. 2007;41(11):1770-81 |
| 359 | 17712043 | Machado M, Bajcar J, Guzzo GC, Einarson TR. Sensitivity of patient outcomes to pharmacist interventions. Part I: systematic review and meta-analysis in diabetes management. Ann Pharmacother. 2007;41(10):1569-82 |
| 363 | 17420201 | Phansalkar S, Hoffman JM, Nebeker JR, Hurdle JF. Pharmacists versus nonpharmacists in adverse drug event detection: a meta-analysis and systematic review. Am J Health Syst Pharm. 2007;64(8):842-9 |
| 371 | 16456206 | Royal S, Smeaton L, Avery AJ, Hurwitz B, Sheikh A. Interventions in primary care to reduce medication related adverse events and hospital admissions: systematic review and meta-analysis. Qual Saf Health Care. 2006;15(1):23-31 |
| 483 | 26755524 | Anthonywalsh K, ORiordan D, Kearney PM, Timmons S, Byrne S. Improving the appropriateness of prescribing in older patients: A systematic review and meta-analysis of pharmacists interventions in secondary care. Age Ageing. 2016;45(2):201-9 |
| 641 | 27660570 | Deichmann RE, Morledge MD, Ulep R, Shaffer JP, Davies P, Van Driel ML. A metaanalysis of interventions to improve adherence to lipid-lowering medication. Ochsner J. 2016;16(3):230-7 |
| 679 |  | Einarson TR, Segal HJ, Mann JL. Pharmacist impact on serum drug level utilization. A meta-analysis. J Soc Adm Pharm. 1989;6(3):109-16 |
| 865 | 28977687 | Khalil H, Bell B, Chambers H, Sheikh A, Avery AJ. Professional, structural and organisational interventions in primary care for reducing medication errors. Cochrane Database Syst Rev. 2017;2017(10): |
| 916 |  | Li X, Mao M, Ping Q. Effect of pharmaceutical care programs on glycemic control in patients with diabetes mellitus: A meta-analysis of randomized controlled trials. J Pharm Technol. 2010;26(5):255-63 |
| 1038 |  | Ni Y, Chen Y, Huang W. The effect of pharmaceutical care programs on blood pressure control in individuals with hypertension: A meta-analysis. J Pharm Technol. 2009;25(5):292-6 |
| 1119 | 30175841 | Rankin A, Cadogan CA, Patterson SM, Kerse N, Cardwell CR, Bradley MC, Ryan C, Hughes C. Interventions to improve the appropriate use of polypharmacy for older people. Cochrane Database Syst Rev. 2018;2018(9): |
| 1353 | 29693291 | Xu R, Xie X, Li S, Chen X, Wang S, Hu C, Lv X. Interventions to improve medication adherence among Chinese patients with hypertension: a systematic review and meta-analysis of randomized controlled trails. Int J Pharm Pract. 2018;26(4):291-301 |
| 1377 | 26963251 | Aguiar PM, Brito GD, Lima TD, Santos A, Lyra DP, Storpirtis S. Investigating Sources of Heterogeneity in Randomized Controlled Trials of the Effects of Pharmacist Interventions on Glycemic Control in Type 2 Diabetic Patients: A Systematic Review and Meta-Analysis. Plos One. 2016;11(3): |
| 1409 | 29590146 | Cheema E, Alhomoud FK, Kinsara A, Alsiddik J, Barnawi MH, Al-Muwallad MA, Abed SA, Elrggal ME, Mohamed MMA. The impact of pharmacists-led medicines reconciliation on healthcare outcomes in secondary care: A systematic review and meta-analysis of randomized controlled trials. Plos One. 2018;13(3): |
| 1417 | 26560139 | Conn VS, Ruppar TM, Chase JAD, Enriquez M, Cooper PS. Interventions to Improve Medication Adherence in Hypertensive Patients: Systematic Review and Meta-analysis. Curr Hypertens Rep. 2015;17(12): |
| 1421 | 29344366 | Crawshaw J, Auyeung V, Ashworth L, Norton S, Weinman J. Healthcare provider-led interventions to support medication adherence following ACS: a meta-analysis. Open Heart. 2017;4(2): |
| 1472 | 27059768 | Johansson T, Abuzahra ME, Keller S, Mann E, Faller B, Sommerauer C, Hock J, Loffler C, Kochling A, Schuler J, Flamm M, Sonnichsen A. Impact of strategies to reduce polypharmacy on clinically relevant endpoints: a systematic review and meta-analysis. Br J Clin Pharmacol. 2016;82(2):532-48 |
| 1490 | 19155168 | Lemmens KMM, Nieboer AP, Huijsman R. A systematic review of integrated use of disease-management interventions in asthma and COPD. Respir Med. 2009;103(5):670-91 |
| 1512 | 27549581 | Mekonnen AB, Abebe TB, McLachlan AJ, Brien JAE. Impact of electronic medication reconciliation interventions on medication discrepancies at hospital transitions: a systematic review and meta-analysis. BMC Med Inform Decis Mak. 2016;16(): |
| 1532 | 28417456 | Normansell R, Kew KM, Stovold E. Interventions to improve adherence to inhaled steroids for asthma. Cochrane Database Syst Rev. 2017;(4): |
| 1540 | 27413005 | Rash JA, Campbell DJT, Tonelli M, Campbell TS. A systematic review of interventions to improve adherence to statin medication: What do we know about what works?. Prev Med. 2016;90():155-69 |
| 1551 | 27317347 | Ruppar TM, Cooper PS, Mehr DR, Delgado JM, Dunbar-Jacob JM. Medication Adherence Interventions Improve Heart Failure Mortality and Readmission Rates: Systematic Review and Meta-Analysis of Controlled Trials. J Am Heart Assoc. 2016;5(6): |
| 1610 | 28483983 | Zhu YC, Zhou YF, Zhang L, Zhang J, Lin J. Efficacy of interventions for adherence to the immunosuppressive therapy in kidney transplant recipients: a meta-analysis and systematic review. J Invest Med. 2017;65(7):1049-56 |
| 2451 | 31097278 | Anand TN, Joseph LM, Geetha AV, Prabhakaran D, Jeemon P. Task sharing with non-physician health-care workers for management of blood pressure in low-income and middle-income countries: a systematic review and meta-analysis. Lancet Glob Health. 2019;7(6):e761-e71 |
| 2495 | 30897055 | Bonetti AF, Reis WC, Mendes AM, Rotta I, Tonin FS, Fernandez-Llimos F, Pontarolo R. Impact of Pharmacist-led Discharge Counseling on Hospital Readmission and Emergency Department Visits: A Systematic Review and Meta-analysis. J Hosp Med. 2019;14():E1-E8 |
| 2527 | 31436877 | Choi YJ, Kim H. Effect of pharmacy-led medication reconciliation in emergency departments: A systematic review and meta-analysis. J Clin Pharm Ther. 2019;(): |
| 2568 | 30707465 | Edwards Z, Ziegler L, Craigs C, Blenkinsopp A, Bennett MI. Pharmacist educational interventions for cancer pain management: a systematic review and meta-analysis. Int J Pharm Pract. 2019;27(4):336-45 |
| 2682 | 31135496 | Lee H, Ryu K, Sohn Y, Kim J, Suh GY, Kim E. Impact on Patient Outcomes of Pharmacist Participation in Multidisciplinary Critical Care Teams: A Systematic Review and Meta-Analysis. Crit Care Med. 2019;47(9):1243-50 |
| 2709 | 31427210 | Lussier ME, Evans HJ, Wright EA, Gionfriddo MR. The impact of community pharmacist involvement on transitions of care: A systematic review and meta-analysis. J Am Pharm Assoc (2003). 2019;(): |
| 2725 | 31102109 | McKay C, Park C, Chang J, Brackbill M, Choi JY, Lee JH, Kim SH. Systematic Review and Meta-analysis of Pharmacist-Led Transitions of Care Services on the 30-Day All-Cause Readmission Rate of Patients with Congestive Heart Failure. Clin Drug Investig. 2019;39(8):703-12 |
| 2747 | 31213852 | Mubarak N, Hatah E, Khan TM, Zin CS. A systematic review and meta-analysis of the impact of collaborative practice between community pharmacist and general practitioner on asthma management. J Asthma Allergy. 2019;12():109-53 |
| 2771 | 31351119 | Parajuli DR, Kourbelis C, Franzon J, Newman P, McKinnon RA, Shakib S, Whitehead D, Clark RA. Effectiveness of the Pharmacist-Involved Multidisciplinary Management of Heart Failure to Improve Hospitalizations and Mortality Rates in 4630 Patients: A Systematic Review and Meta-Analysis of Randomized Controlled Trials. J Card Fail. 2019;25(9):744-56 |
| 2786 | 30685443 | Presley B, Groot W, Pavlova M. Pharmacy-led interventions to improve medication adherence among adults with diabetes: A systematic review and meta-analysis. Res Social Adm Pharm. 2019;15(9):1057-67 |
| 2822 | 30698721 | Saha SK, Hawes L, Mazza D. Effectiveness of interventions involving pharmacists on antibiotic prescribing by general practitioners: a systematic review and meta-analysis. J Antimicrob Chemother. 2019;74(5):1173-81 |
| 2852 | 31431337 | Snoswell CL. A meta-analysis of pharmacists and pharmacy technicians accuracy checking proficiency. Res Social Adm Pharm. 2019;(): |
| 2870 | 30920431 | Tasai S, Kumpat N, Dilokthornsakul P, Chaiyakunapruk N, Saini B, Dhippayom T. Impact of Medication Reviews Delivered by Community Pharmacist to Elderly Patients on Polypharmacy: A Meta-analysis of Randomized Controlled Trials. J Patient Saf. 2019;(): |
| 2922 | 31225940 | Yuan C, Ding Y, Zhou K, Huang Y, Xi X. Clinical outcomes of community pharmacy services: A systematic review and meta-analysis. Health Soc Care Community. 2019;27(5):e567-e87 |
| 2933 |  | Babar ZUD, Kousar R, Hasan SS, Scahill S, Curley LE. Glycemic control through pharmaceutical care: a meta-analysis of randomized controlled trials. Journal of Pharmaceutical Health Services Research. 2019;10(1):35-44 |
| 3003 | 32419932 | Zhang J, Li X, Xie J, Zheng W. Evaluation of a clinical pharmacist consultation service for patients with infectious diseases in China: A systematic review and meta-analysis. European Journal of Hospital Pharmacy. 2019;(): |
| 2382 | 31941489 | Abbott, R. A., Moore, D. A., Rogers, M., Bethel, A., Stein, K. and Coon, J. T.. Effectiveness of pharmacist home visits for individuals at risk of medication-related problems: a systematic review and meta-analysis of randomised controlled trials. BMC Health Serv Res. 2020;20(1):39 |
| 2386 | 31777082 | Alshehri, A. A., Jalal, Z., Cheema, E., Haque, M. S., Jenkins, D. and Yahyouche, A.. Impact of the pharmacist-led intervention on the control of medical cardiovascular risk factors for the primary prevention of cardiovascular disease in general practice: A systematic review and meta-analysis of randomised controlled trials. Br J Clin Pharmacol. 2020;86(1): 29-38 |
| 2415 | 31868236 | Brown, J. V. E., Walton, N., Meader, N., Todd, A., Webster, L. A. D., Steele, R., Sampson, S. J., Churchill, R., McMillan, D., Gilbody, S. and Ekers, D.. Pharmacy-based management for depression in adults. Cochrane Database Syst Rev. 2019;2019(12): |
| 2433 | 31684695 | Carson-Chahhoud, K. V., Livingstone-Banks, J., Sharrad, K. J., Kopsaftis, Z., Brinn, M. P., To-A-nan, R. and Bond, C. M.. Community pharmacy personnel interventions for smoking cessation. Cochrane Database Syst Rev. 2019;2019(10): |
| 2390 | 31465121 | Lee, S. W. H., Mak, V. S. L. and Tang, Y. W.. Pharmacist services in nursing homes: A systematic review and meta-analysis. Br J Clin Pharmacol. 2019;85(12): 2668-2688 |
| 2444 | 31711390 | Martinez-Mardones, F., Fernandez-Llimos, F., Benrimoj, S. I., Ahumada-Canale, A., Plaza-Plaza, J. C., Tonin, F. S. and Garcia-Cardenas, V.. Systematic Review and Meta-Analysis of Medication Reviews Conducted by Pharmacists on Cardiovascular Diseases Risk Factors in Ambulatory Care. J Am Heart Assoc. 2019;8(22): |
| 2443 | 31758656 | Mizokami, F., Mizuno, T., Kanamori, K., Oyama, S., Nagamatsu, T., Lee, J. K. and Kobayashi, T.. Clinical medication review type III of polypharmacy reduced unplanned hospitalizations in older adults: A meta-analysis of randomized clinical trials. Geriatrics & Gerontology International. 2020;(): |
| 2384 | 31919801 | Tecklenborg, S., Byrne, C., Cahir, C., Brown, L. and Bennett, K.. Interventions to Reduce Adverse Drug Event-Related Outcomes in Older Adults: A Systematic Review and Meta-analysis. Drugs Aging. 2020;37(2): 91-98 |
| 10135 | 32418821 | Dixon DL, Khaddage S, Bhagat S, Koenig RA, Salgado TM, Baker WL. Effect of pharmacist interventions on reducing low-density lipoprotein cholesterol (LDL-C) levels: A systematic review and meta-analysis. J Clin Lipidol. 2020;14(3):282-92 e4 |
| 10147 | 32107837 | Jia X, Zhou S, Luo D, Zhao X, Zhou Y, Cui YM. Effect of pharmacist-led interventions on medication adherence and inhalation technique in adult patients with asthma or COPD: A systematic review and meta-analysis. J Clin Pharm Ther. 2020;45(5):904-17 |
| 10125 | 32487066 | Lin G, Huang R, Zhang J, Li G, Chen L, Xi X. Clinical and economic outcomes of hospital pharmaceutical care: a systematic review and meta-analysis. BMC Health Serv Res. 2020;20(1):487 |
| 10095 | 33240478 | Manias E, Kusljic S, Wu A. Interventions to reduce medication errors in adult medical and surgical settings: a systematic review. Ther Adv Drug Saf. 2020;11():2042098620968309 |
| 10139 | 32328958 | Naseralallah LM, Hussain TA, Jaam M, Pawluk SA. Impact of pharmacist interventions on medication errors in hospitalized pediatric patients: a systematic review and meta-analysis. Int J Clin Pharm. 2020;42(4):979-94 |
| 10092 | 33294063 | Nassur PL, Forgerini M, Mastroianni PC, Lucchetta RC. Clinical pharmacy services in Brazil, particularly cardiometabolic diseases: a systematic scoping review and meta-analyses. Pharm Pract (Granada). 2020;18(4):2131 |
| 10154 | 32022107 | Nogueira M, Otuyama LJ, Rocha PA, Pinto VB. Pharmaceutical care-based interventions in type 2 diabetes mellitus : a systematic review and meta-analysis of randomized clinical trials. Einstein (Sao Paulo). 2020;18():eRW4686 |
| 10149 | 32078109 | Qin SB, Zhang XY, Fu Y, Nie XY, Liu J, Shi LW, Cui YM. The impact of the clinical pharmacist-led interventions in China: A systematic review and Meta-Analysis. Int J Clin Pharm. 2020;42(2):366-77 |
| 10093 | 33270710 | Singh-Franco D, Mastropietro DR, Metzner M, Dressler MD, Fares A, Johnson M, De La Rosa D, Wolowich WR. Impact of pharmacy-supported interventions on proportion of patients receiving non-indicated acid suppressive therapy upon discharge: A systematic review and meta-analysis. PLoS One. 2020;15(12):e0243134 |
| 10121 | 32629653 | Yi ZM, Li TT, Tang QY, Zhang Y, Willis S, Zhai SD. Content and impact of pharmacy services for patients with Parkinsons disease: A systematic review and meta-analysis. Medicine (Baltimore). 2020;99(27):e20758 |
| 10023 | 34353754 | Ahmed A, Abdulelah Dujaili J, Rehman IU, Lay Hong AC, Hashmi FK, Awaisu A, Chaiyakunapruk N. Effect of pharmacist care on clinical outcomes among people living with HIV/AIDS: A systematic review and meta-analysis. Res Social Adm Pharm. 2021;(): |
| 10046 | 33965357 | Al-Babtain B, Cheema E, Hadi MA. Impact of community-pharmacist-led medication review programmes on patient outcomes: A systematic review and meta-analysis of randomised controlled trials. Res Social Adm Pharm. 2021;(): |
| 10713 |  | Arunmanakul P, Kengkla K, Chaiyasothi T, Phrommintikul A, Ruengorn C, Permsuwan U, Thakkinstian A, Page RL, II, Munger MA, Nathisuwan S, Chaiyakunapruk N. Effects of pharmacist interventions on heart failure outcomes: A systematic review and meta-analysis. JACCP JAm Coll Clin Pharm. 2021;4(7):871-82 |
| 10017 | 34459265 | Bunchuailua W, Samprasit N, Kotirum S, Kapol N. Impact of Pharmacist Activities in Patients With Depression: A Systematic Review and Meta-analysis of Randomized Controlled Trials. Ann Pharmacother. 2021;():10600280211041274 |
| 10761 | 28671909 | De Oliveira GS, Castro-Alves LJ, Kendall MC, McCarthy R. Effectiveness of Pharmacist Intervention to Reduce Medication Errors and Health-Care Resources Utilization after Transitions of Care: A Meta-analysis of Randomized Controlled Trials. J Patient Saf. 2021;17(5):375-80 |
| 10027 | 34303610 | Foot H, Scott I, Sturman N, Whitty JA, Rixon K, Connelly L, Williams I, Freeman C. Impact of pharmacist and physician collaborations in primary care on reducing readmission to hospital: A systematic review and meta-analysis. Res Social Adm Pharm. 2021;(): |
| 11005 | 34228962 | Hernandez MH, Juanes-Borrego AM, Mila R, Mangues-Bafalluy MA, Mestres C. The Impact of Pharmaceutical Care in Multidisciplinary Teams on Health Outcomes: Systematic Review and Meta-Analysis. J Am Med Dir Assoc. 2021;22(12):2518-26 |
| 10033 | 34161388 | Jaam M, Naseralallah LM, Hussain TA, Pawluk SA. Pharmacist-led educational interventions provided to healthcare providers to reduce medication errors: A systematic review and meta-analysis. PLoS One. 2021;16(6):e0253588 |
| 10063 | 33678564 | Kelly WN, Ho MJ, Bullers K, Klocksieben F, Kumar A. Association of pharmacist counseling with adherence, 30-day readmission, and mortality: A systematic review and meta-analysis of randomized trials. J Am Pharm Assoc (2003). 2021;61(3):340-50 e5 |
| 10008 | 34716722 | Lee SY, An SH. Impact of pharmacist intervention in antibiotic stewardship programmes for critically ill neonates: A systematic review and meta-analysis. J Clin Pharm Ther. 2021;(): |
| 10054 | 33817821 | Mahdavi H, Esmaily H. Impact of educational intervention by community pharmacists on asthma clinical outcomes, quality of life and medication adherence: A systematic review and meta-analysis. J Clin Pharm Ther. 2021;46(5):1254-62 |
| 10029 | 34287846 | Marcum ZA, Jiang S, Bacci JL, Ruppar TM. Pharmacist-led interventions to improve medication adherence in older adults: A meta-analysis. J Am Geriatr Soc. 2021;69(11):3301-11 |
| 10844 |  | Memisoglu M, Çelik Y. Meta-analysis of pharmacist-led and pharmacist-physician intervention on blood pressure control. Ital J Med. 2021;15(3):145-53 |
| 10040 | 34047881 | Murray E, Bieniek K, Del Aguila M, Egodage S, Litzinger S, Mazouz A, Mills H, Liska J. Impact of pharmacy intervention on influenza vaccination acceptance: a systematic literature review and meta-analysis. Int J Clin Pharm. 2021;43(5):1163-72 |
| 10105 | 32949161 | Nakanishi M, Mizuno T, Mizokami F, Koseki T, Takahashi K, Tsuboi N, Katz M, Lee JK, Yamada S. Impact of pharmacist intervention for blood pressure control in patients with chronic kidney disease: A meta-analysis of randomized clinical trials. J Clin Pharm Ther. 2021;46(1):114-20 |
| 10109 | 32881191 | Oñatibia-Astibia A, Malet-Larrea A, Gastelurrutia M, Calvo B, Goyenechea E. Community pharmacist interventions to improve adherence to lipid lowering medication and their influence on clinical outcomes: A systematic review and meta-analysis. J Eval Clin Pract. 2021;27(2):451-63 |
| 10032 | 34180129 | Powell RE, Zaccardi F, Beebe C, Chen XM, Crawford A, Cuddeback J, Gabbay RA, Kissela L, Litchman ML, Mehta R, Meneghini L, Pantalone KM, Rajpathak S, Scribner P, Skelley JW, Khunti K. Strategies for overcoming therapeutic inertia in type 2 diabetes: A systematic review and meta-analysis. Diabetes Obes Metab. 2021;23(9):2137-54 |
| 10030 | 34240570 | Schumacher PM, Becker N, Tsuyuki RT, Griese-Mammen N, Koshman SL, McDonald MA, Bouvy M, Rutten FH, Laufs U, Böhm M, Schulz M. The evidence for pharmacist care in outpatients with heart failure: a systematic review and meta-analysis. ESC Heart Fail. 2021;8(5):3566-76 |
| 10076 | 33486825 | Thapa P, Lee SWH, Kc B, Dujaili JA, Mohamed Ibrahim MI, Gyawali S. Pharmacist-led intervention on chronic pain management: A systematic review and meta-analysis. Br J Clin Pharmacol. 2021;87(8):3028-42 |
| 10003 | 34848531 | Mahmood RK, Gillani SW, Alzaabi MJ, Gulam SM. Evaluation of inappropriate antibiotic prescribing and management through pharmacist-led antimicrobial stewardship programmes: a meta-analysis of evidence. Eur J Hosp Pharm. 2022;29(1):2-7 |

**S5. Bradford’s analysis of the journal scattering (n=501) of the 2012 articles included in the 138 meta-analysis**


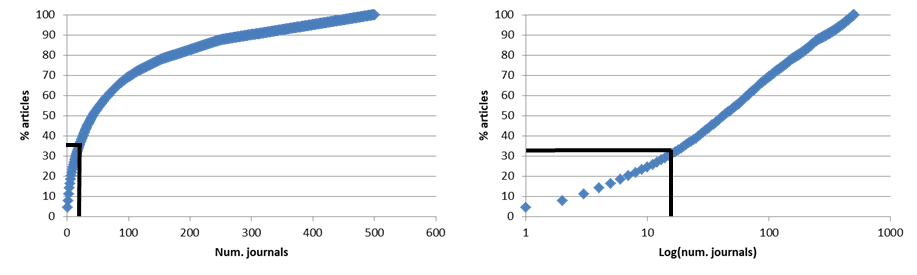


**S6. Top 20 most productive journals according to the included 2021 articles**

|  | Num. | Percentage | Cumulative percentage |
| --- | --- | --- | --- |
| Am J Health Syst Pharm | 92 | 4.6 | 4.6 |
| Pharmacotherapy | 69 | 3.4 | 8.0 |
| Ann Pharmacother | 67 | 3.3 | 11.3 |
| J Am Pharm Assoc (2003) | 58 | 2.9 | 14.2 |
| Int J Clin Pharm | 43 | 2.1 | 16.4 |
| Arch Intern Med | 42 | 2.1 | 18.4 |
| Am J Hosp Pharm | 38 | 1.9 | 20.3 |
| J Clin Pharm Ther | 30 | 1.5 | 21.8 |
| Patient Educ Couns | 30 | 1.5 | 23.3 |
| J Am Geriatr Soc | 26 | 1.3 | 24.6 |
| J Am Pharm Assoc (Wash)* | 25 | 1.2 | 25.8 |
| BMJ | 24 | 1.2 | 27.0 |
| J Manag Care Pharm | 23 | 1.1 | 28.2 |
| Am J Manag Care | 20 | 1.0 | 29.2 |
| Br J Clin Pharmacol | 20 | 1.0 | 30.2 |
| Pharm World Sci** | 20 | 1.0 | 31.2 |
| J Gen Intern Med | 19 | 0.9 | 32.1 |
| PLoS One | 19 | 0.9 | 33.1 |
| Am J Med | 18 | 0.9 | 33.9 |
| J Acquir Immune Defic Syndr | 18 | 0.9 | 34.8 |
| * Former title of J Am Pharm Assoc (2003)  ** Former title of Int J Clin Pharm | | | |

**S7. List of 2012 articles included in the 138 meta-analyses and presenting MeSH (with the frequency of appearance in meta-analyses)**

| **PMID** | **Freq** |
| --- | --- |
| 15745726 | 15 |
| 15839185 | 15 |
| 17298190 | 15 |
| 12495164 | 14 |
| 15522983 | 14 |
| 17057049 | 14 |
| 22971205 | 14 |
| 8610730 | 13 |
| 9782691 | 13 |
| 14583903 | 13 |
| 15793171 | 13 |
| 18815843 | 13 |
| 19401477 | 13 |
| 19433702 | 13 |
| 19552750 | 13 |
| 20394460 | 13 |
| 22672148 | 13 |
| 11232739 | 12 |
| 12816022 | 12 |
| 16791717 | 12 |
| 17387123 | 12 |
| 17452390 | 12 |
| 23161124 | 12 |
| 25301007 | 12 |
| 8833557 | 11 |
| 8840744 | 11 |
| 11322670 | 11 |
| 12482009 | 11 |
| 15665005 | 11 |
| 16423096 | 11 |
| 17101639 | 11 |
| 17523968 | 11 |
| 19966072 | 11 |
| 21335480 | 11 |
| 24247275 | 11 |
| 25234932 | 11 |
| 25420946 | 11 |
| 8076871 | 10 |
| 11079287 | 10 |
| 11443021 | 10 |
| 11714206 | 10 |
| 16042672 | 10 |
| 16647628 | 10 |
| 17502632 | 10 |
| 21143256 | 10 |
| 23023841 | 10 |
| 24327590 | 10 |
| 507073 | 9 |
| 10417035 | 9 |
| 10493325 | 9 |
| 11130223 | 9 |
| 12587810 | 9 |
| 12847034 | 9 |
| 14524649 | 9 |
| 16534045 | 9 |
| 17251316 | 9 |
| 17824872 | 9 |
| 18094011 | 9 |
| 18401223 | 9 |
| 19289342 | 9 |
| 20809276 | 9 |
| 25243680 | 9 |
| 9824519 | 8 |
| 15011764 | 8 |
| 15085948 | 8 |
| 15186866 | 8 |
| 15701763 | 8 |
| 16623018 | 8 |
| 16905764 | 8 |
| 17959576 | 8 |
| 22021025 | 8 |
| 22101426 | 8 |
| 25789518 | 8 |
| 26499503 | 8 |
| 28448779 | 8 |
| 4201656 | 7 |
| 11486980 | 7 |
| 11601666 | 7 |
| 11739221 | 7 |
| 15121348 | 7 |
| 15599837 | 7 |
| 17493184 | 7 |
| 18577730 | 7 |
| 18684418 | 7 |
| 20059474 | 7 |
| 20212029 | 7 |
| 20929988 | 7 |
| 21365405 | 7 |
| 22751755 | 7 |
| 24117908 | 7 |
| 24293339 | 7 |
| 6693703 | 6 |
| 7547154 | 6 |
| 9399611 | 6 |
| 9605777 | 6 |
| 9782692 | 6 |
| 10772372 | 6 |
| 12492615 | 6 |
| 12851665 | 6 |
| 14514935 | 6 |
| 15827075 | 6 |
| 15903284 | 6 |
| 15991756 | 6 |
| 19843062 | 6 |
| 19933962 | 6 |
| 20817685 | 6 |
| 21209140 | 6 |
| 21392837 | 6 |
| 21534640 | 6 |
| 21733413 | 6 |
| 21987530 | 6 |
| 22520532 | 6 |
| 22797831 | 6 |
| 23537456 | 6 |
| 23810267 | 6 |
| 23975474 | 6 |
| 24241260 | 6 |
| 24943987 | 6 |
| 2195066 | 5 |
| 10683133 | 5 |
| 10730020 | 5 |
| 10974963 | 5 |
| 11372906 | 5 |
| 11402636 | 5 |
| 11790365 | 5 |
| 12350190 | 5 |
| 14584229 | 5 |
| 15040645 | 5 |
| 15257726 | 5 |
| 16945057 | 5 |
| 18285347 | 5 |
| 19029501 | 5 |
| 19388074 | 5 |
| 21119098 | 5 |
| 22083873 | 5 |
| 23219937 | 5 |
| 23821088 | 5 |
| 24030130 | 5 |
| 24530144 | 5 |
| 25347723 | 5 |
| 26434752 | 5 |
| 29723341 | 5 |
| 9017775 | 4 |
| 10547170 | 4 |
| 10897509 | 4 |
| 11034037 | 4 |
| 11176549 | 4 |
| 11298072 | 4 |
| 12921497 | 4 |
| 14687321 | 4 |
| 14748894 | 4 |
| 15006588 | 4 |
| 15385274 | 4 |
| 15465619 | 4 |
| 15655101 | 4 |
| 15767225 | 4 |
| 15812103 | 4 |
| 16137215 | 4 |
| 16219893 | 4 |
| 16290975 | 4 |
| 16334075 | 4 |
| 16342302 | 4 |
| 16880458 | 4 |
| 16916809 | 4 |
| 17056608 | 4 |
| 17272285 | 4 |
| 18302840 | 4 |
| 18585510 | 4 |
| 18834368 | 4 |
| 18848176 | 4 |
| 20040704 | 4 |
| 20180606 | 4 |
| 20492060 | 4 |
| 20513826 | 4 |
| 21220657 | 4 |
| 21320198 | 4 |
| 22028418 | 4 |
| 22175269 | 4 |
| 22292061 | 4 |
| 22548691 | 4 |
| 22570370 | 4 |
| 23023840 | 4 |
| 23033226 | 4 |
| 23137954 | 4 |
| 23408163 | 4 |
| 23463811 | 4 |
| 23677794 | 4 |
| 23677816 | 4 |
| 24002278 | 4 |
| 24321403 | 4 |
| 24515550 | 4 |
| 24798710 | 4 |
| 25085406 | 4 |
| 25135805 | 4 |
| 25315207 | 4 |
| 25562774 | 4 |
| 26376830 | 4 |
| 27058907 | 4 |
| 27087103 | 4 |
| 28438152 | 4 |
| 28449205 | 4 |
| 31129917 | 4 |
| 73694 | 3 |
| 369673 | 3 |
| 430732 | 3 |
| 937876 | 3 |
| 1173830 | 3 |
| 1608408 | 3 |
| 1770472 | 3 |
| 2012087 | 3 |
| 2072772 | 3 |
| 2188495 | 3 |
| 3941533 | 3 |
| 3958683 | 3 |
| 6377291 | 3 |
| 6426377 | 3 |
| 6624755 | 3 |
| 6757394 | 3 |
| 7595265 | 3 |
| 8123955 | 3 |
| 8564589 | 3 |
| 8739813 | 3 |
| 9165562 | 3 |
| 9640480 | 3 |
| 9701098 | 3 |
| 9717348 | 3 |
| 9825420 | 3 |
| 10256913 | 3 |
| 10271922 | 3 |
| 10422996 | 3 |
| 10709370 | 3 |
| 10852081 | 3 |
| 10892822 | 3 |
| 10916893 | 3 |
| 10930970 | 3 |
| 11182878 | 3 |
| 11329521 | 3 |
| 11411442 | 3 |
| 11440296 | 3 |
| 11485129 | 3 |
| 11678959 | 3 |
| 11990212 | 3 |
| 12038949 | 3 |
| 12185826 | 3 |
| 12360171 | 3 |
| 12389879 | 3 |
| 12688435 | 3 |
| 12695272 | 3 |
| 12704485 | 3 |
| 12749524 | 3 |
| 12841801 | 3 |
| 12882844 | 3 |
| 12925422 | 3 |
| 14565841 | 3 |
| 15098638 | 3 |
| 15151791 | 3 |
| 15191244 | 3 |
| 15372866 | 3 |
| 15479780 | 3 |
| 15485755 | 3 |
| 15546536 | 3 |
| 15730115 | 3 |
| 15730122 | 3 |
| 15811483 | 3 |
| 15888822 | 3 |
| 15903283 | 3 |
| 15915277 | 3 |
| 16036495 | 3 |
| 16331115 | 3 |
| 16466329 | 3 |
| 16522892 | 3 |
| 16858241 | 3 |
| 17042984 | 3 |
| 17061966 | 3 |
| 17121506 | 3 |
| 17202024 | 3 |
| 17249858 | 3 |
| 17286786 | 3 |
| 17365206 | 3 |
| 17404187 | 3 |
| 17461708 | 3 |
| 17616493 | 3 |
| 17703369 | 3 |
| 17823108 | 3 |
| 17880478 | 3 |
| 18093505 | 3 |
| 18318852 | 3 |
| 18574016 | 3 |
| 18626028 | 3 |
| 18679820 | 3 |
| 18721222 | 3 |
| 19001531 | 3 |
| 19189907 | 3 |
| 19389122 | 3 |
| 19497643 | 3 |
| 19583673 | 3 |
| 19649720 | 3 |
| 19884620 | 3 |
| 19933963 | 3 |
| 19947803 | 3 |
| 20008582 | 3 |
| 20178395 | 3 |
| 20360753 | 3 |
| 20653352 | 3 |
| 20798988 | 3 |
| 20856132 | 3 |
| 21326328 | 3 |
| 21354671 | 3 |
| 21384066 | 3 |
| 21385240 | 3 |
| 21418100 | 3 |
| 21508941 | 3 |
| 21649843 | 3 |
| 21732195 | 3 |
| 21744191 | 3 |
| 21787031 | 3 |
| 22068326 | 3 |
| 22080794 | 3 |
| 22246660 | 3 |
| 22380642 | 3 |
| 22388601 | 3 |
| 22621795 | 3 |
| 22679128 | 3 |
| 22866361 | 3 |
| 22930542 | 3 |
| 22937977 | 3 |
| 23224336 | 3 |
| 23270495 | 3 |
| 23340148 | 3 |
| 23537457 | 3 |
| 23636160 | 3 |
| 23709583 | 3 |
| 23786500 | 3 |
| 23984830 | 3 |
| 24505112 | 3 |
| 24598584 | 3 |
| 24657991 | 3 |
| 24671049 | 3 |
| 24726204 | 3 |
| 24814043 | 3 |
| 24868083 | 3 |
| 24895001 | 3 |
| 24914089 | 3 |
| 25193393 | 3 |
| 25403606 | 3 |
| 25479285 | 3 |
| 25590930 | 3 |
| 25634012 | 3 |
| 25774017 | 3 |
| 25917376 | 3 |
| 26063762 | 3 |
| 26201426 | 3 |
| 26297239 | 3 |
| 26755665 | 3 |
| 26803086 | 3 |
| 26883526 | 3 |
| 27452158 | 3 |
| 27473571 | 3 |
| 27605543 | 3 |
| 29295829 | 3 |
| 29379953 | 3 |
| 30819640 | 3 |
| 701704 | 2 |
| 930938 | 2 |
| 1554003 | 2 |
| 1570874 | 2 |
| 1735279 | 2 |
| 2092306 | 2 |
| 2096208 | 2 |
| 3706334 | 2 |
| 3769774 | 2 |
| 3967574 | 2 |
| 3968925 | 2 |
| 3974333 | 2 |
| 4019790 | 2 |
| 6167606 | 2 |
| 6638026 | 2 |
| 6732373 | 2 |
| 6773971 | 2 |
| 6843196 | 2 |
| 6987865 | 2 |
| 7081241 | 2 |
| 7215889 | 2 |
| 7698064 | 2 |
| 8013609 | 2 |
| 8166034 | 2 |
| 8602380 | 2 |
| 9069692 | 2 |
| 9100140 | 2 |
| 9184710 | 2 |
| 9230143 | 2 |
| 9278202 | 2 |
| 9475816 | 2 |
| 9786807 | 2 |
| 9853633 | 2 |
| 9855341 | 2 |
| 9931569 | 2 |
| 10051255 | 2 |
| 10246655 | 2 |
| 10254211 | 2 |
| 10256898 | 2 |
| 10283978 | 2 |
| 10371363 | 2 |
| 10404919 | 2 |
| 10587835 | 2 |
| 10600085 | 2 |
| 10609444 | 2 |
| 10772374 | 2 |
| 10935668 | 2 |
| 10948785 | 2 |
| 11059437 | 2 |
| 11079286 | 2 |
| 11091772 | 2 |
| 11343005 | 2 |
| 11372905 | 2 |
| 11387182 | 2 |
| 11471478 | 2 |
| 11560201 | 2 |
| 11576977 | 2 |
| 11693075 | 2 |
| 11755291 | 2 |
| 11785996 | 2 |
| 11821367 | 2 |
| 11823086 | 2 |
| 11826512 | 2 |
| 11918503 | 2 |
| 11936711 | 2 |
| 11978169 | 2 |
| 12066964 | 2 |
| 12070114 | 2 |
| 12140806 | 2 |
| 12148711 | 2 |
| 12234455 | 2 |
| 12269709 | 2 |
| 12480116 | 2 |
| 12688433 | 2 |
| 12710553 | 2 |
| 12722963 | 2 |
| 12781934 | 2 |
| 12788301 | 2 |
| 12789875 | 2 |
| 12850874 | 2 |
| 12889503 | 2 |
| 12957333 | 2 |
| 14503113 | 2 |
| 14504113 | 2 |
| 14558182 | 2 |
| 14573327 | 2 |
| 14613471 | 2 |
| 14618079 | 2 |
| 14626750 | 2 |
| 14687254 | 2 |
| 15018255 | 2 |
| 15031417 | 2 |
| 15066200 | 2 |
| 15095516 | 2 |
| 15145971 | 2 |
| 15302014 | 2 |
| 15310688 | 2 |
| 15338843 | 2 |
| 15476999 | 2 |
| 15492340 | 2 |
| 15642765 | 2 |
| 15767226 | 2 |
| 15841891 | 2 |
| 15843283 | 2 |
| 15868754 | 2 |
| 15893183 | 2 |
| 15899730 | 2 |
| 15948086 | 2 |
| 15985049 | 2 |
| 15997043 | 2 |
| 16046491 | 2 |
| 16096888 | 2 |
| 16219894 | 2 |
| 16300420 | 2 |
| 16305300 | 2 |
| 16452523 | 2 |
| 16529338 | 2 |
| 16595786 | 2 |
| 16602223 | 2 |
| 16624687 | 2 |
| 16765240 | 2 |
| 16809238 | 2 |
| 16809752 | 2 |
| 16882872 | 2 |
| 16945056 | 2 |
| 16960256 | 2 |
| 17004019 | 2 |
| 17085982 | 2 |
| 17138505 | 2 |
| 17309907 | 2 |
| 17320942 | 2 |
| 17353570 | 2 |
| 17389290 | 2 |
| 17533206 | 2 |
| 17658958 | 2 |
| 17846552 | 2 |
| 17875113 | 2 |
| 18056946 | 2 |
| 18164894 | 2 |
| 18192127 | 2 |
| 18192132 | 2 |
| 18192260 | 2 |
| 18211656 | 2 |
| 18317424 | 2 |
| 18359731 | 2 |
| 18359733 | 2 |
| 18363528 | 2 |
| 18654123 | 2 |
| 18719760 | 2 |
| 18776075 | 2 |
| 18796426 | 2 |
| 18928735 | 2 |
| 18947262 | 2 |
| 19043801 | 2 |
| 19139220 | 2 |
| 19174696 | 2 |
| 19193572 | 2 |
| 19210281 | 2 |
| 19220069 | 2 |
| 19318602 | 2 |
| 19398689 | 2 |
| 19433997 | 2 |
| 19477398 | 2 |
| 19633917 | 2 |
| 19675319 | 2 |
| 19685933 | 2 |
| 19694865 | 2 |
| 19721382 | 2 |
| 19808372 | 2 |
| 19848566 | 2 |
| 19890086 | 2 |
| 20002510 | 2 |
| 20031834 | 2 |
| 20051135 | 2 |
| 20100808 | 2 |
| 20102876 | 2 |
| 20207925 | 2 |
| 20209303 | 2 |
| 20371056 | 2 |
| 20429739 | 2 |
| 20616786 | 2 |
| 20734138 | 2 |
| 20817937 | 2 |
| 20823391 | 2 |
| 20858878 | 2 |
| 20862544 | 2 |
| 20883060 | 2 |
| 20920322 | 2 |
| 21077956 | 2 |
| 21092191 | 2 |
| 21307692 | 2 |
| 21318595 | 2 |
| 21335296 | 2 |
| 21394570 | 2 |
| 21428465 | 2 |
| 21442282 | 2 |
| 21511454 | 2 |
| 21544559 | 2 |
| 21621947 | 2 |
| 21721962 | 2 |
| 21774100 | 2 |
| 21798784 | 2 |
| 21806770 | 2 |
| 21834113 | 2 |
| 21865093 | 2 |
| 21911633 | 2 |
| 21916908 | 2 |
| 22048929 | 2 |
| 22165359 | 2 |
| 22216772 | 2 |
| 22230825 | 2 |
| 22392419 | 2 |
| 22471165 | 2 |
| 22491014 | 2 |
| 22618978 | 2 |
| 22656403 | 2 |
| 22810890 | 2 |
| 22821790 | 2 |
| 23130391 | 2 |
| 23277421 | 2 |
| 23403978 | 2 |
| 23437933 | 2 |
| 23531984 | 2 |
| 23617687 | 2 |
| 23636151 | 2 |
| 23636155 | 2 |
| 23690938 | 2 |
| 23811030 | 2 |
| 23830534 | 2 |
| 23869999 | 2 |
| 24024587 | 2 |
| 24057434 | 2 |
| 24195894 | 2 |
| 24259595 | 2 |
| 24259651 | 2 |
| 24269619 | 2 |
| 24570297 | 2 |
| 24676715 | 2 |
| 24712335 | 2 |
| 24733770 | 2 |
| 24993458 | 2 |
| 25022824 | 2 |
| 25128076 | 2 |
| 25142870 | 2 |
| 25147171 | 2 |
| 25174019 | 2 |
| 25187228 | 2 |
| 25187339 | 2 |
| 25257687 | 2 |
| 25278326 | 2 |
| 25322157 | 2 |
| 25358723 | 2 |
| 25470782 | 2 |
| 25486932 | 2 |
| 25557203 | 2 |
| 25742062 | 2 |
| 25811824 | 2 |
| 25880360 | 2 |
| 25881226 | 2 |
| 25889580 | 2 |
| 25985395 | 2 |
| 25991594 | 2 |
| 26011547 | 2 |
| 26156431 | 2 |
| 26190132 | 2 |
| 26289950 | 2 |
| 26302142 | 2 |
| 26451051 | 2 |
| 26455537 | 2 |
| 26527048 | 2 |
| 26580349 | 2 |
| 26695700 | 2 |
| 26857999 | 2 |
| 27062272 | 2 |
| 27108410 | 2 |
| 27195696 | 2 |
| 27311911 | 2 |
| 27385237 | 2 |
| 27414973 | 2 |
| 27578624 | 2 |
| 27582175 | 2 |
| 27875542 | 2 |
| 27915457 | 2 |
| 28025928 | 2 |
| 28107122 | 2 |
| 28445474 | 2 |
| 29121408 | 2 |
| 29625912 | 2 |
| 29672567 | 2 |
| 29754251 | 2 |
| 29877609 | 2 |
| 30655090 | 2 |
| 31590926 | 2 |
| 31659776 | 2 |
| 47833 | 1 |
| 67805 | 1 |
| 90371 | 1 |
| 91901 | 1 |
| 111548 | 1 |
| 212774 | 1 |
| 287930 | 1 |
| 350043 | 1 |
| 484566 | 1 |
| 490882 | 1 |
| 500572 | 1 |
| 525645 | 1 |
| 623120 | 1 |
| 644633 | 1 |
| 645716 | 1 |
| 657069 | 1 |
| 660211 | 1 |
| 665683 | 1 |
| 700622 | 1 |
| 761334 | 1 |
| 793389 | 1 |
| 821723 | 1 |
| 837099 | 1 |
| 879182 | 1 |
| 902005 | 1 |
| 906995 | 1 |
| 911007 | 1 |
| 1089237 | 1 |
| 1319935 | 1 |
| 1392755 | 1 |
| 1506947 | 1 |
| 1554946 | 1 |
| 1576393 | 1 |
| 1610514 | 1 |
| 1614233 | 1 |
| 1815972 | 1 |
| 1824793 | 1 |
| 1882873 | 1 |
| 1895265 | 1 |
| 1905438 | 1 |
| 1928139 | 1 |
| 1934001 | 1 |
| 2003499 | 1 |
| 2023020 | 1 |
| 2031691 | 1 |
| 2127324 | 1 |
| 2200380 | 1 |
| 2228629 | 1 |
| 2290770 | 1 |
| 2404053 | 1 |
| 2461148 | 1 |
| 2497587 | 1 |
| 2504064 | 1 |
| 2569323 | 1 |
| 2571354 | 1 |
| 2595518 | 1 |
| 2695999 | 1 |
| 2745106 | 1 |
| 2899491 | 1 |
| 2921441 | 1 |
| 2975558 | 1 |
| 3047691 | 1 |
| 3087162 | 1 |
| 3126652 | 1 |
| 3146893 | 1 |
| 3235485 | 1 |
| 3428141 | 1 |
| 3449560 | 1 |
| 3492181 | 1 |
| 3498877 | 1 |
| 3503941 | 1 |
| 3575028 | 1 |
| 3631108 | 1 |
| 3731255 | 1 |
| 3879962 | 1 |
| 3976675 | 1 |
| 4006739 | 1 |
| 4081405 | 1 |
| 4277505 | 1 |
| 4352958 | 1 |
| 4498247 | 1 |
| 4733411 | 1 |
| 4808929 | 1 |
| 4829072 | 1 |
| 5135190 | 1 |
| 6153057 | 1 |
| 6171080 | 1 |
| 6350633 | 1 |
| 6406886 | 1 |
| 6418647 | 1 |
| 6422381 | 1 |
| 6430689 | 1 |
| 6507445 | 1 |
| 6545038 | 1 |
| 6742256 | 1 |
| 6745092 | 1 |
| 6794364 | 1 |
| 6848154 | 1 |
| 6908909 | 1 |
| 7053953 | 1 |
| 7059257 | 1 |
| 7105976 | 1 |
| 7109741 | 1 |
| 7114339 | 1 |
| 7148856 | 1 |
| 7211873 | 1 |
| 7246556 | 1 |
| 7282695 | 1 |
| 7283555 | 1 |
| 7325182 | 1 |
| 7328538 | 1 |
| 7351109 | 1 |
| 7373255 | 1 |
| 7436639 | 1 |
| 7487258 | 1 |
| 7565975 | 1 |
| 7570406 | 1 |
| 7575800 | 1 |
| 7594930 | 1 |
| 7613410 | 1 |
| 7656118 | 1 |
| 7696720 | 1 |
| 7711346 | 1 |
| 7720757 | 1 |
| 7746762 | 1 |
| 7767193 | 1 |
| 7796721 | 1 |
| 7852669 | 1 |
| 7885976 | 1 |
| 7942724 | 1 |
| 7977921 | 1 |
| 8026662 | 1 |
| 8028691 | 1 |
| 8078528 | 1 |
| 8108894 | 1 |
| 8114050 | 1 |
| 8135233 | 1 |
| 8147544 | 1 |
| 8160687 | 1 |
| 8170427 | 1 |
| 8179443 | 1 |
| 8227238 | 1 |
| 8231604 | 1 |
| 8263895 | 1 |
| 8347901 | 1 |
| 8475211 | 1 |
| 8480784 | 1 |
| 8499815 | 1 |
| 8501992 | 1 |
| 8538891 | 1 |
| 8659554 | 1 |
| 8737434 | 1 |
| 8780347 | 1 |
| 8809280 | 1 |
| 8824077 | 1 |
| 8873488 | 1 |
| 8933299 | 1 |
| 8971306 | 1 |
| 9008249 | 1 |
| 9054234 | 1 |
| 9083713 | 1 |
| 9085319 | 1 |
| 9136321 | 1 |
| 9184706 | 1 |
| 9192927 | 1 |
| 9218921 | 1 |
| 9248601 | 1 |
| 9248604 | 1 |
| 9250230 | 1 |
| 9250560 | 1 |
| 9268276 | 1 |
| 9269394 | 1 |
| 9277247 | 1 |
| 9373814 | 1 |
| 9377207 | 1 |
| 9389427 | 1 |
| 9425799 | 1 |
| 9437477 | 1 |
| 9467435 | 1 |
| 9533058 | 1 |
| 9545151 | 1 |
| 9606452 | 1 |
| 9640129 | 1 |
| 9663635 | 1 |
| 9663636 | 1 |
| 9679795 | 1 |
| 9689193 | 1 |
| 9740348 | 1 |
| 9742976 | 1 |
| 9763719 | 1 |
| 9782686 | 1 |
| 9782690 | 1 |
| 9872695 | 1 |
| 9872700 | 1 |
| 9917434 | 1 |
| 9930041 | 1 |
| 9930042 | 1 |
| 10027665 | 1 |
| 10086671 | 1 |
| 10116059 | 1 |
| 10119188 | 1 |
| 10119735 | 1 |
| 10135148 | 1 |
| 10135646 | 1 |
| 10138706 | 1 |
| 10171854 | 1 |
| 10186470 | 1 |
| 10191559 | 1 |
| 10193067 | 1 |
| 10208788 | 1 |
| 10219280 | 1 |
| 10221375 | 1 |
| 10231256 | 1 |
| 10242843 | 1 |
| 10247477 | 1 |
| 10247885 | 1 |
| 10258767 | 1 |
| 10263894 | 1 |
| 10264741 | 1 |
| 10267933 | 1 |
| 10268102 | 1 |
| 10268504 | 1 |
| 10269317 | 1 |
| 10272531 | 1 |
| 10306928 | 1 |
| 10307387 | 1 |
| 10315745 | 1 |
| 10378621 | 1 |
| 10383547 | 1 |
| 10387383 | 1 |
| 10404930 | 1 |
| 10451079 | 1 |
| 10465325 | 1 |
| 10478992 | 1 |
| 10533347 | 1 |
| 10547171 | 1 |
| 10560719 | 1 |
| 10588620 | 1 |
| 10599010 | 1 |
| 10609971 | 1 |
| 10610013 | 1 |
| 10623052 | 1 |
| 10630654 | 1 |
| 10664641 | 1 |
| 10671804 | 1 |
| 10671912 | 1 |
| 10674779 | 1 |
| 10678300 | 1 |
| 10683131 | 1 |
| 10691817 | 1 |
| 10730019 | 1 |
| 10730021 | 1 |
| 10730025 | 1 |
| 10783940 | 1 |
| 10790817 | 1 |
| 10796491 | 1 |
| 10796542 | 1 |
| 10805048 | 1 |
| 10807190 | 1 |
| 10877496 | 1 |
| 10886462 | 1 |
| 10908471 | 1 |
| 10939559 | 1 |
| 10941955 | 1 |
| 10955751 | 1 |
| 10980076 | 1 |
| 10999500 | 1 |
| 11034043 | 1 |
| 11039967 | 1 |
| 11111356 | 1 |
| 11115952 | 1 |
| 11148942 | 1 |
| 11206689 | 1 |
| 11213861 | 1 |
| 11264761 | 1 |
| 11279717 | 1 |
| 11286147 | 1 |
| 11297338 | 1 |
| 11302353 | 1 |
| 11304773 | 1 |
| 11310518 | 1 |
| 11341138 | 1 |
| 11349751 | 1 |
| 11372911 | 1 |
| 11382368 | 1 |
| 11406063 | 1 |
| 11446129 | 1 |
| 11450926 | 1 |
| 11471481 | 1 |
| 11486981 | 1 |
| 11493372 | 1 |
| 11502614 | 1 |
| 11510252 | 1 |
| 11560203 | 1 |
| 11564356 | 1 |
| 11579986 | 1 |
| 11603236 | 1 |
| 11669361 | 1 |
| 11679387 | 1 |
| 11715828 | 1 |
| 11819185 | 1 |
| 11837562 | 1 |
| 11851640 | 1 |
| 11887408 | 1 |
| 11897728 | 1 |
| 11910714 | 1 |
| 11936740 | 1 |
| 12020141 | 1 |
| 12020186 | 1 |
| 12021683 | 1 |
| 12023501 | 1 |
| 12027926 | 1 |
| 12028174 | 1 |
| 12066966 | 1 |
| 12073859 | 1 |
| 12076376 | 1 |
| 12161054 | 1 |
| 12185830 | 1 |
| 12383144 | 1 |
| 12383573 | 1 |
| 12394555 | 1 |
| 12399344 | 1 |
| 12407485 | 1 |
| 12409135 | 1 |
| 12431277 | 1 |
| 12435467 | 1 |
| 12455304 | 1 |
| 12455306 | 1 |
| 12464839 | 1 |
| 12469544 | 1 |
| 12494151 | 1 |
| 12502654 | 1 |
| 12527148 | 1 |
| 12549814 | 1 |
| 12549816 | 1 |
| 12549944 | 1 |
| 12570110 | 1 |
| 12570118 | 1 |
| 12578516 | 1 |
| 12587811 | 1 |
| 12622605 | 1 |
| 12624628 | 1 |
| 12635454 | 1 |
| 12644142 | 1 |
| 12659057 | 1 |
| 12663573 | 1 |
| 12668691 | 1 |
| 12671103 | 1 |
| 12671780 | 1 |
| 12683743 | 1 |
| 12698766 | 1 |
| 12756157 | 1 |
| 12875612 | 1 |
| 12882845 | 1 |
| 12892032 | 1 |
| 12898489 | 1 |
| 12917891 | 1 |
| 12971668 | 1 |
| 12975221 | 1 |
| 14504627 | 1 |
| 14506507 | 1 |
| 14509861 | 1 |
| 14521770 | 1 |
| 14526208 | 1 |
| 14581257 | 1 |
| 14583918 | 1 |
| 14617500 | 1 |
| 14626747 | 1 |
| 14626756 | 1 |
| 14631476 | 1 |
| 14632964 | 1 |
| 14641395 | 1 |
| 14656614 | 1 |
| 14692370 | 1 |
| 14726370 | 1 |
| 14768768 | 1 |
| 14769157 | 1 |
| 14998595 | 1 |
| 15001596 | 1 |
| 15013038 | 1 |
| 15017487 | 1 |
| 15023226 | 1 |
| 15040540 | 1 |
| 15040652 | 1 |
| 15086645 | 1 |
| 15121349 | 1 |
| 15123479 | 1 |
| 15139843 | 1 |
| 15147529 | 1 |
| 15161077 | 1 |
| 15177526 | 1 |
| 15210260 | 1 |
| 15213488 | 1 |
| 15249261 | 1 |
| 15252415 | 1 |
| 15282232 | 1 |
| 15287235 | 1 |
| 15297305 | 1 |
| 15298236 | 1 |
| 15313938 | 1 |
| 15328395 | 1 |
| 15333056 | 1 |
| 15338855 | 1 |
| 15363008 | 1 |
| 15366436 | 1 |
| 15369427 | 1 |
| 15372834 | 1 |
| 15372868 | 1 |
| 15380493 | 1 |
| 15446781 | 1 |
| 15466769 | 1 |
| 15527158 | 1 |
| 15546454 | 1 |
| 15557360 | 1 |
| 15562220 | 1 |
| 15563363 | 1 |
| 15566021 | 1 |
| 15577269 | 1 |
| 15611506 | 1 |
| 15636668 | 1 |
| 15642874 | 1 |
| 15646702 | 1 |
| 15649331 | 1 |
| 15659884 | 1 |
| 15669583 | 1 |
| 15675553 | 1 |
| 15690118 | 1 |
| 15718178 | 1 |
| 15745893 | 1 |
| 15751766 | 1 |
| 15762903 | 1 |
| 15769772 | 1 |
| 15823192 | 1 |
| 15841895 | 1 |
| 15842446 | 1 |
| 15860094 | 1 |
| 15861266 | 1 |
| 15867495 | 1 |
| 15869555 | 1 |
| 15911723 | 1 |
| 15928222 | 1 |
| 15928265 | 1 |
| 15940125 | 1 |
| 15950838 | 1 |
| 15961289 | 1 |
| 15966780 | 1 |
| 15990127 | 1 |
| 15993559 | 1 |
| 16010172 | 1 |
| 16019102 | 1 |
| 16030370 | 1 |
| 16055616 | 1 |
| 16088280 | 1 |
| 16105344 | 1 |
| 16107494 | 1 |
| 16107989 | 1 |
| 16128502 | 1 |
| 16129870 | 1 |
| 16186262 | 1 |
| 16188750 | 1 |
| 16191135 | 1 |
| 16194132 | 1 |
| 16236850 | 1 |
| 16243954 | 1 |
| 16249712 | 1 |
| 16264046 | 1 |
| 16277797 | 1 |
| 16278621 | 1 |
| 16280273 | 1 |
| 16283835 | 1 |
| 16287200 | 1 |
| 16288070 | 1 |
| 16373466 | 1 |
| 16420109 | 1 |
| 16430351 | 1 |
| 16433927 | 1 |
| 16435071 | 1 |
| 16437191 | 1 |
| 16449396 | 1 |
| 16469469 | 1 |
| 16476115 | 1 |
| 16513919 | 1 |
| 16542130 | 1 |
| 16569793 | 1 |
| 16611656 | 1 |
| 16643574 | 1 |
| 16647616 | 1 |
| 16652320 | 1 |
| 16673835 | 1 |
| 16674782 | 1 |
| 16684940 | 1 |
| 16686585 | 1 |
| 16708043 | 1 |
| 16735965 | 1 |
| 16754526 | 1 |
| 16763491 | 1 |
| 16770291 | 1 |
| 16796760 | 1 |
| 16816027 | 1 |
| 16824155 | 1 |
| 16835670 | 1 |
| 16839250 | 1 |
| 16908918 | 1 |
| 16914379 | 1 |
| 16989688 | 1 |
| 16999646 | 1 |
| 16999661 | 1 |
| 17011901 | 1 |
| 17056206 | 1 |
| 17062324 | 1 |
| 17066294 | 1 |
| 17069104 | 1 |
| 17081285 | 1 |
| 17089238 | 1 |
| 17116767 | 1 |
| 17129333 | 1 |
| 17142247 | 1 |
| 17149995 | 1 |
| 17166985 | 1 |
| 17189585 | 1 |
| 17192116 | 1 |
| 17224714 | 1 |
| 17224844 | 1 |
| 17227325 | 1 |
| 17239062 | 1 |
| 17244788 | 1 |
| 17244879 | 1 |
| 17261454 | 1 |
| 17261462 | 1 |
| 17263651 | 1 |
| 17263930 | 1 |
| 17272798 | 1 |
| 17284503 | 1 |
| 17299180 | 1 |
| 17322163 | 1 |
| 17338480 | 1 |
| 17339106 | 1 |
| 17440008 | 1 |
| 17443097 | 1 |
| 17460126 | 1 |
| 17488138 | 1 |
| 17510001 | 1 |
| 17510030 | 1 |
| 17510038 | 1 |
| 17518010 | 1 |
| 17533211 | 1 |
| 17534463 | 1 |
| 17538003 | 1 |
| 17542760 | 1 |
| 17545346 | 1 |
| 17557211 | 1 |
| 17581436 | 1 |
| 17587364 | 1 |
| 17588243 | 1 |
| 17605569 | 1 |
| 17605699 | 1 |
| 17609051 | 1 |
| 17617501 | 1 |
| 17645463 | 1 |
| 17687061 | 1 |
| 17692494 | 1 |
| 17693890 | 1 |
| 17708080 | 1 |
| 17724358 | 1 |
| 17802984 | 1 |
| 17804302 | 1 |
| 17823105 | 1 |
| 17846938 | 1 |
| 17848420 | 1 |
| 17850292 | 1 |
| 17891043 | 1 |
| 17893418 | 1 |
| 17901162 | 1 |
| 17913440 | 1 |
| 17915147 | 1 |
| 17954806 | 1 |
| 17985009 | 1 |
| 17986517 | 1 |
| 18031971 | 1 |
| 18083668 | 1 |
| 18084874 | 1 |
| 18088263 | 1 |
| 18089983 | 1 |
| 18090049 | 1 |
| 18091554 | 1 |
| 18154470 | 1 |
| 18160008 | 1 |
| 18165755 | 1 |
| 18206449 | 1 |
| 18238771 | 1 |
| 18268174 | 1 |
| 18271430 | 1 |
| 18283077 | 1 |
| 18299546 | 1 |
| 18302808 | 1 |
| 18319393 | 1 |
| 18326962 | 1 |
| 18332291 | 1 |
| 18337052 | 1 |
| 18340537 | 1 |
| 18345369 | 1 |
| 18351194 | 1 |
| 18351473 | 1 |
| 18356607 | 1 |
| 18360186 | 1 |
| 18373136 | 1 |
| 18377134 | 1 |
| 18473697 | 1 |
| 18474492 | 1 |
| 18493615 | 1 |
| 18568696 | 1 |
| 18577762 | 1 |
| 18584303 | 1 |
| 18601636 | 1 |
| 18613991 | 1 |
| 18631255 | 1 |
| 18645509 | 1 |
| 18653816 | 1 |
| 18677064 | 1 |
| 18693777 | 1 |
| 18714113 | 1 |
| 18718886 | 1 |
| 18752691 | 1 |
| 18769349 | 1 |
| 18790668 | 1 |
| 18792423 | 1 |
| 18801781 | 1 |
| 18807252 | 1 |
| 18936700 | 1 |
| 18954244 | 1 |
| 18973713 | 1 |
| 19001195 | 1 |
| 19006440 | 1 |
| 19010447 | 1 |
| 19018919 | 1 |
| 19019805 | 1 |
| 19032015 | 1 |
| 19032017 | 1 |
| 19093922 | 1 |
| 19106728 | 1 |
| 19125548 | 1 |
| 19129262 | 1 |
| 19139786 | 1 |
| 19145785 | 1 |
| 19171611 | 1 |
| 19174529 | 1 |
| 19188371 | 1 |
| 19191134 | 1 |
| 19203773 | 1 |
| 19212017 | 1 |
| 19214120 | 1 |
| 19219923 | 1 |
| 19233107 | 1 |
| 19241731 | 1 |
| 19254484 | 1 |
| 19256406 | 1 |
| 19258441 | 1 |
| 19281937 | 1 |
| 19289346 | 1 |
| 19300114 | 1 |
| 19300186 | 1 |
| 19318600 | 1 |
| 19327616 | 1 |
| 19327625 | 1 |
| 19328598 | 1 |
| 19336646 | 1 |
| 19345053 | 1 |
| 19348923 | 1 |
| 19357068 | 1 |
| 19365719 | 1 |
| 19366977 | 1 |
| 19367427 | 1 |
| 19375519 | 1 |
| 19401125 | 1 |
| 19417112 | 1 |
| 19443169 | 1 |
| 19465470 | 1 |
| 19479782 | 1 |
| 19512945 | 1 |
| 19527383 | 1 |
| 19531961 | 1 |
| 19557400 | 1 |
| 19566401 | 1 |
| 19566950 | 1 |
| 19620163 | 1 |
| 19623570 | 1 |
| 19651405 | 1 |
| 19652618 | 1 |
| 19656386 | 1 |
| 19659407 | 1 |
| 19674217 | 1 |
| 19687522 | 1 |
| 19694112 | 1 |
| 19694337 | 1 |
| 19710251 | 1 |
| 19713556 | 1 |
| 19717798 | 1 |
| 19729401 | 1 |
| 19748331 | 1 |
| 19771504 | 1 |
| 19776297 | 1 |
| 19785675 | 1 |
| 19786674 | 1 |
| 19808281 | 1 |
| 19811593 | 1 |
| 19812099 | 1 |
| 19815286 | 1 |
| 19817937 | 1 |
| 19843492 | 1 |
| 19854632 | 1 |
| 19858047 | 1 |
| 19858174 | 1 |
| 19858812 | 1 |
| 19864187 | 1 |
| 19896021 | 1 |
| 19911481 | 1 |
| 19918632 | 1 |
| 19923314 | 1 |
| 19933787 | 1 |
| 19939445 | 1 |
| 19942984 | 1 |
| 19948623 | 1 |
| 19952780 | 1 |
| 19959322 | 1 |
| 19962676 | 1 |
| 19962679 | 1 |
| 19995493 | 1 |
| 20009091 | 1 |
| 20014180 | 1 |
| 20019345 | 1 |
| 20028960 | 1 |
| 20040705 | 1 |
| 20044067 | 1 |
| 20044368 | 1 |
| 20048680 | 1 |
| 20052529 | 1 |
| 20074348 | 1 |
| 20082055 | 1 |
| 20137091 | 1 |
| 20146111 | 1 |
| 20148619 | 1 |
| 20170329 | 1 |
| 20192941 | 1 |
| 20199989 | 1 |
| 20200284 | 1 |
| 20201628 | 1 |
| 20208066 | 1 |
| 20222036 | 1 |
| 20233982 | 1 |
| 20298399 | 1 |
| 20350694 | 1 |
| 20374349 | 1 |
| 20381924 | 1 |
| 20383572 | 1 |
| 20394511 | 1 |
| 20413121 | 1 |
| 20429738 | 1 |
| 20464350 | 1 |
| 20495161 | 1 |
| 20500277 | 1 |
| 20500732 | 1 |
| 20524710 | 1 |
| 20532194 | 1 |
| 20534872 | 1 |
| 20535847 | 1 |
| 20538819 | 1 |
| 20539854 | 1 |
| 20552377 | 1 |
| 20569973 | 1 |
| 20575654 | 1 |
| 20589438 | 1 |
| 20595717 | 1 |
| 20598745 | 1 |
| 20619448 | 1 |
| 20636651 | 1 |
| 20640949 | 1 |
| 20640958 | 1 |
| 20646898 | 1 |
| 20650358 | 1 |
| 20658830 | 1 |
| 20679589 | 1 |
| 20683336 | 1 |
| 20701874 | 1 |
| 20724395 | 1 |
| 20816187 | 1 |
| 20824548 | 1 |
| 20841521 | 1 |
| 20852438 | 1 |
| 20866164 | 1 |
| 20890160 | 1 |
| 20945114 | 1 |
| 20956707 | 1 |
| 20957423 | 1 |
| 21070222 | 1 |
| 21070296 | 1 |
| 21071074 | 1 |
| 21080835 | 1 |
| 21092015 | 1 |
| 21106046 | 1 |
| 21119101 | 1 |
| 21119344 | 1 |
| 21122501 | 1 |
| 21139446 | 1 |
| 21144064 | 1 |
| 21153694 | 1 |
| 21182355 | 1 |
| 21196451 | 1 |
| 21204963 | 1 |
| 21211865 | 1 |
| 21247578 | 1 |
| 21271406 | 1 |
| 21287904 | 1 |
| 21295198 | 1 |
| 21300712 | 1 |
| 21308343 | 1 |
| 21311842 | 1 |
| 21315219 | 1 |
| 21317163 | 1 |
| 21323566 | 1 |
| 21366642 | 1 |
| 21384285 | 1 |
| 21386021 | 1 |
| 21390262 | 1 |
| 21401764 | 1 |
| 21419684 | 1 |
| 21424272 | 1 |
| 21434698 | 1 |
| 21452051 | 1 |
| 21457056 | 1 |
| 21459309 | 1 |
| 21473965 | 1 |
| 21476945 | 1 |
| 21478777 | 1 |
| 21545614 | 1 |
| 21605732 | 1 |
| 21605733 | 1 |
| 21627699 | 1 |
| 21636796 | 1 |
| 21643784 | 1 |
| 21647425 | 1 |
| 21649623 | 1 |
| 21653307 | 1 |
| 21683161 | 1 |
| 21688350 | 1 |
| 21704360 | 1 |
| 21706311 | 1 |
| 21710194 | 1 |
| 21715653 | 1 |
| 21725628 | 1 |
| 21729274 | 1 |
| 21729849 | 1 |
| 21732897 | 1 |
| 21733046 | 1 |
| 21737312 | 1 |
| 21747013 | 1 |
| 21788632 | 1 |
| 21791439 | 1 |
| 21791543 | 1 |
| 21802271 | 1 |
| 21806766 | 1 |
| 21830265 | 1 |
| 21849052 | 1 |
| 21864625 | 1 |
| 21890753 | 1 |
| 21896463 | 1 |
| 21906278 | 1 |
| 21923456 | 1 |
| 21930640 | 1 |
| 21931077 | 1 |
| 21956192 | 1 |
| 21982663 | 1 |
| 22000272 | 1 |
| 22033742 | 1 |
| 22045925 | 1 |
| 22053315 | 1 |
| 22076516 | 1 |
| 22094303 | 1 |
| 22095578 | 1 |
| 22106461 | 1 |
| 22123798 | 1 |
| 22134934 | 1 |
| 22137609 | 1 |
| 22151410 | 1 |
| 22171103 | 1 |
| 22180556 | 1 |
| 22196835 | 1 |
| 22211669 | 1 |
| 22230826 | 1 |
| 22231970 | 1 |
| 22232107 | 1 |
| 22236835 | 1 |
| 22249412 | 1 |
| 22282154 | 1 |
| 22283360 | 1 |
| 22296762 | 1 |
| 22298194 | 1 |
| 22335395 | 1 |
| 22339141 | 1 |
| 22343209 | 1 |
| 22345421 | 1 |
| 22356599 | 1 |
| 22357106 | 1 |
| 22362718 | 1 |
| 22382482 | 1 |
| 22382484 | 1 |
| 22382886 | 1 |
| 22395249 | 1 |
| 22398670 | 1 |
| 22410103 | 1 |
| 22424001 | 1 |
| 22435835 | 1 |
| 22455402 | 1 |
| 22486226 | 1 |
| 22488512 | 1 |
| 22498687 | 1 |
| 22523190 | 1 |
| 22527264 | 1 |
| 22535491 | 1 |
| 22548692 | 1 |
| 22554973 | 1 |
| 22570108 | 1 |
| 22572916 | 1 |
| 22628369 | 1 |
| 22652012 | 1 |
| 22665103 | 1 |
| 22679303 | 1 |
| 22680786 | 1 |
| 22688429 | 1 |
| 22691240 | 1 |
| 22736737 | 1 |
| 22761079 | 1 |
| 22762857 | 1 |
| 22774352 | 1 |
| 22784926 | 1 |
| 22785264 | 1 |
| 22790614 | 1 |
| 22821310 | 1 |
| 22830539 | 1 |
| 22849787 | 1 |
| 22901955 | 1 |
| 22924464 | 1 |
| 23007693 | 1 |
| 23017533 | 1 |
| 23021790 | 1 |
| 23033123 | 1 |
| 23046818 | 1 |
| 23050578 | 1 |
| 23067910 | 1 |
| 23113628 | 1 |
| 23113630 | 1 |
| 23171659 | 1 |
| 23184352 | 1 |
| 23195913 | 1 |
| 23197844 | 1 |
| 23216534 | 1 |
| 23217036 | 1 |
| 23225167 | 1 |
| 23229961 | 1 |
| 23229963 | 1 |
| 23229976 | 1 |
| 23244257 | 1 |
| 23248342 | 1 |
| 23251355 | 1 |
| 23261899 | 1 |
| 23263797 | 1 |
| 23273227 | 1 |
| 23281228 | 1 |
| 23282122 | 1 |
| 23324512 | 1 |
| 23332093 | 1 |
| 23337300 | 1 |
| 23337369 | 1 |
| 23352524 | 1 |
| 23368423 | 1 |
| 23378537 | 1 |
| 23383705 | 1 |
| 23390210 | 1 |
| 23391845 | 1 |
| 23418704 | 1 |
| 23418812 | 1 |
| 23462636 | 1 |
| 23508601 | 1 |
| 23514242 | 1 |
| 23537153 | 1 |
| 23539943 | 1 |
| 23541131 | 1 |
| 23553347 | 1 |
| 23561463 | 1 |
| 23568228 | 1 |
| 23581450 | 1 |
| 23630403 | 1 |
| 23636159 | 1 |
| 23640349 | 1 |
| 23644816 | 1 |
| 23656751 | 1 |
| 23670112 | 1 |
| 23689927 | 1 |
| 23696699 | 1 |
| 23697476 | 1 |
| 23706653 | 1 |
| 23708882 | 1 |
| 23715759 | 1 |
| 23719885 | 1 |
| 23759395 | 1 |
| 23771450 | 1 |
| 23784163 | 1 |
| 23800333 | 1 |
| 23812892 | 1 |
| 23813851 | 1 |
| 23819827 | 1 |
| 23848476 | 1 |
| 23904145 | 1 |
| 23908549 | 1 |
| 23910580 | 1 |
| 23918485 | 1 |
| 23928167 | 1 |
| 23948623 | 1 |
| 23950967 | 1 |
| 23956341 | 1 |
| 23964617 | 1 |
| 23971019 | 1 |
| 23975860 | 1 |
| 23988600 | 1 |
| 24006873 | 1 |
| 24007452 | 1 |
| 24007766 | 1 |
| 24021286 | 1 |
| 24024759 | 1 |
| 24052204 | 1 |
| 24062215 | 1 |
| 24065784 | 1 |
| 24073620 | 1 |
| 24095088 | 1 |
| 24116922 | 1 |
| 24185429 | 1 |
| 24225332 | 1 |
| 24237752 | 1 |
| 24238397 | 1 |
| 24248037 | 1 |
| 24259652 | 1 |
| 24279284 | 1 |
| 24289059 | 1 |
| 24313446 | 1 |
| 24322961 | 1 |
| 24331203 | 1 |
| 24334947 | 1 |
| 24352179 | 1 |
| 24375606 | 1 |
| 24385269 | 1 |
| 24388126 | 1 |
| 24399262 | 1 |
| 24438924 | 1 |
| 24445732 | 1 |
| 24449960 | 1 |
| 24456315 | 1 |
| 24473490 | 1 |
| 24473676 | 1 |
| 24513419 | 1 |
| 24521593 | 1 |
| 24565620 | 1 |
| 24566877 | 1 |
| 24586465 | 1 |
| 24651641 | 1 |
| 24675394 | 1 |
| 24687289 | 1 |
| 24705022 | 1 |
| 24708232 | 1 |
| 24733137 | 1 |
| 24733153 | 1 |
| 24733354 | 1 |
| 24759957 | 1 |
| 24766179 | 1 |
| 24771706 | 1 |
| 24773329 | 1 |
| 24779524 | 1 |
| 24785136 | 1 |
| 24803381 | 1 |
| 24847095 | 1 |
| 24913003 | 1 |
| 24917185 | 1 |
| 24930425 | 1 |
| 24969964 | 1 |
| 24981605 | 1 |
| 25027255 | 1 |
| 25046061 | 1 |
| 25062783 | 1 |
| 25087039 | 1 |
| 25089310 | 1 |
| 25105284 | 1 |
| 25153537 | 1 |
| 25166287 | 1 |
| 25166290 | 1 |
| 25216877 | 1 |
| 25250655 | 1 |
| 25277510 | 1 |
| 25295834 | 1 |
| 25329386 | 1 |
| 25350546 | 1 |
| 25364817 | 1 |
| 25409898 | 1 |
| 25451909 | 1 |
| 25456778 | 1 |
| 25469880 | 1 |
| 25516044 | 1 |
| 25539358 | 1 |
| 25546163 | 1 |
| 25550587 | 1 |
| 25560773 | 1 |
| 25594919 | 1 |
| 25595280 | 1 |
| 25617215 | 1 |
| 25628008 | 1 |
| 25631224 | 1 |
| 25664620 | 1 |
| 25732548 | 1 |
| 25733343 | 1 |
| 25742320 | 1 |
| 25744762 | 1 |
| 25753453 | 1 |
| 25755034 | 1 |
| 25764140 | 1 |
| 25805647 | 1 |
| 25817472 | 1 |
| 25820164 | 1 |
| 25833666 | 1 |
| 25872641 | 1 |
| 25873621 | 1 |
| 25885807 | 1 |
| 25910787 | 1 |
| 25919315 | 1 |
| 25952653 | 1 |
| 25953807 | 1 |
| 25988589 | 1 |
| 25991591 | 1 |
| 25991595 | 1 |
| 25991596 | 1 |
| 26003154 | 1 |
| 26003155 | 1 |
| 26009557 | 1 |
| 26032843 | 1 |
| 26092965 | 1 |
| 26095656 | 1 |
| 26161487 | 1 |
| 26163876 | 1 |
| 26179814 | 1 |
| 26187183 | 1 |
| 26198433 | 1 |
| 26199142 | 1 |
| 26207363 | 1 |
| 26214111 | 1 |
| 26277573 | 1 |
| 26362420 | 1 |
| 26377496 | 1 |
| 26406772 | 1 |
| 26415743 | 1 |
| 26416996 | 1 |
| 26522838 | 1 |
| 26544202 | 1 |
| 26553894 | 1 |
| 26573754 | 1 |
| 26590989 | 1 |
| 26597401 | 1 |
| 26645932 | 1 |
| 26647412 | 1 |
| 26669645 | 1 |
| 26681443 | 1 |
| 26697487 | 1 |
| 26702083 | 1 |
| 26702612 | 1 |
| 26766552 | 1 |
| 26786346 | 1 |
| 26802916 | 1 |
| 26845640 | 1 |
| 26852290 | 1 |
| 26861468 | 1 |
| 26911532 | 1 |
| 26916590 | 1 |
| 26922904 | 1 |
| 26961826 | 1 |
| 27038405 | 1 |
| 27229333 | 1 |
| 27230437 | 1 |
| 27253380 | 1 |
| 27254036 | 1 |
| 27255463 | 1 |
| 27255504 | 1 |
| 27283244 | 1 |
| 27298211 | 1 |
| 27304922 | 1 |
| 27306856 | 1 |
| 27352414 | 1 |
| 27364808 | 1 |
| 27365262 | 1 |
| 27385197 | 1 |
| 27398874 | 1 |
| 27415585 | 1 |
| 27432017 | 1 |
| 27449546 | 1 |
| 27456328 | 1 |
| 27475032 | 1 |
| 27488552 | 1 |
| 27578187 | 1 |
| 27579824 | 1 |
| 27606938 | 1 |
| 27714762 | 1 |
| 27742628 | 1 |
| 27759828 | 1 |
| 27777076 | 1 |
| 27815524 | 1 |
| 27825313 | 1 |
| 27864732 | 1 |
| 27866336 | 1 |
| 27890050 | 1 |
| 27890885 | 1 |
| 27931556 | 1 |
| 27943247 | 1 |
| 27955991 | 1 |
| 27969054 | 1 |
| 28003248 | 1 |
| 28004259 | 1 |
| 28076735 | 1 |
| 28179250 | 1 |
| 28184045 | 1 |
| 28212652 | 1 |
| 28219367 | 1 |
| 28302636 | 1 |
| 28334979 | 1 |
| 28349576 | 1 |
| 28357623 | 1 |
| 28385023 | 1 |
| 28447542 | 1 |
| 28453819 | 1 |
| 28472499 | 1 |
| 28477678 | 1 |
| 28478718 | 1 |
| 28505367 | 1 |
| 28549592 | 1 |
| 28550698 | 1 |
| 28581821 | 1 |
| 28596021 | 1 |
| 28600913 | 1 |
| 28601691 | 1 |
| 28622739 | 1 |
| 28622740 | 1 |
| 28639465 | 1 |
| 28646377 | 1 |
| 28666310 | 1 |
| 28669290 | 1 |
| 28698169 | 1 |
| 28705697 | 1 |
| 28717929 | 1 |
| 28745260 | 1 |
| 28746708 | 1 |
| 28793906 | 1 |
| 28796597 | 1 |
| 28804870 | 1 |
| 28826670 | 1 |
| 28852915 | 1 |
| 28891153 | 1 |
| 28965748 | 1 |
| 28975305 | 1 |
| 28986515 | 1 |
| 28991599 | 1 |
| 28993287 | 1 |
| 29040609 | 1 |
| 29050887 | 1 |
| 29083969 | 1 |
| 29097094 | 1 |
| 29100199 | 1 |
| 29104008 | 1 |
| 29153444 | 1 |
| 29162290 | 1 |
| 29169856 | 1 |
| 29196825 | 1 |
| 29209863 | 1 |
| 29241617 | 1 |
| 29249774 | 1 |
| 29265040 | 1 |
| 29267800 | 1 |
| 29300205 | 1 |
| 29331037 | 1 |
| 29384024 | 1 |
| 29396511 | 1 |
| 29418054 | 1 |
| 29437537 | 1 |
| 29438607 | 1 |
| 29439095 | 1 |
| 29478012 | 1 |
| 29603074 | 1 |
| 29603353 | 1 |
| 29606624 | 1 |
| 29694290 | 1 |
| 29715303 | 1 |
| 29720459 | 1 |
| 29768945 | 1 |
| 29778344 | 1 |
| 29808711 | 1 |
| 29846666 | 1 |
| 29884657 | 1 |
| 30022334 | 1 |
| 30030308 | 1 |
| 30036160 | 1 |
| 30051222 | 1 |
| 30058987 | 1 |
| 30083727 | 1 |
| 30088321 | 1 |
| 30099887 | 1 |
| 30126891 | 1 |
| 30150128 | 1 |
| 30170967 | 1 |
| 30205184 | 1 |
| 30214718 | 1 |
| 30225268 | 1 |
| 30261980 | 1 |
| 30343615 | 1 |
| 30403432 | 1 |
| 30465630 | 1 |
| 30514376 | 1 |
| 30531528 | 1 |
| 30608549 | 1 |
| 30641686 | 1 |
| 30669851 | 1 |
| 30689745 | 1 |
| 30729723 | 1 |
| 30739389 | 1 |
| 30932898 | 1 |
| 30953062 | 1 |
| 30957240 | 1 |
| 30975119 | 1 |
| 31067214 | 1 |
| 31234840 | 1 |
| 31240722 | 1 |
| 31287736 | 1 |
| 31413839 | 1 |
| 31466535 | 1 |
| 31650506 | 1 |
| 31685404 | 1 |
| 31732440 | 1 |
| 31761141 | 1 |
| 31778428 | 1 |
| 31798894 | 1 |
| 31866388 | 1 |
| 31883204 | 1 |
| 31907396 | 1 |
| 31955987 | 1 |
| 32011966 | 1 |
| 32339716 | 1 |
| 32524511 | 1 |
| 32562716 | 1 |
| 32611807 | 1 |
| 32820591 | 1 |
| 32857651 | 1 |
| 32861616 | 1 |
| 32967439 | 1 |
| 33055227 | 1 |
| 33580553 | 1 |

**S8. Distribution of the MeSH assigned per article over time**


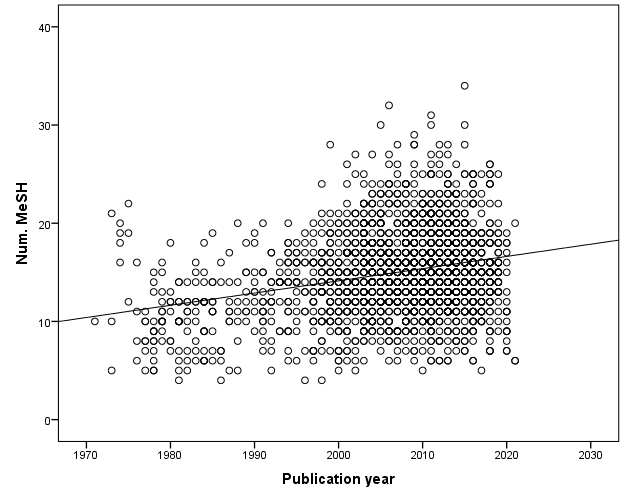


**S9. Distribution of Major MeSH assigned assigned per article over time**


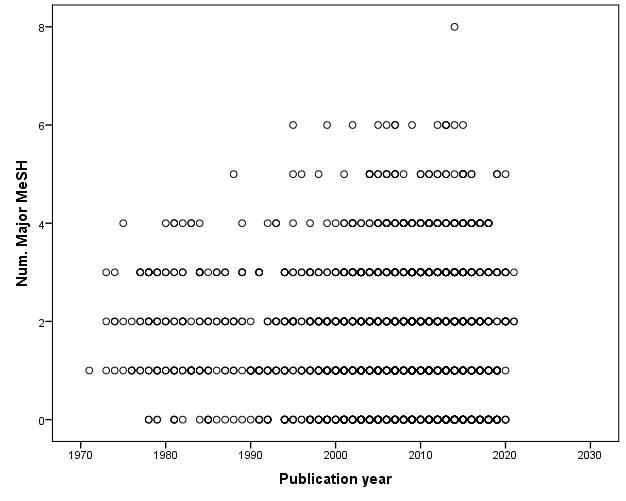


**S10. Percentage of MeSH terms classified as Major MeSH in the articles over time**


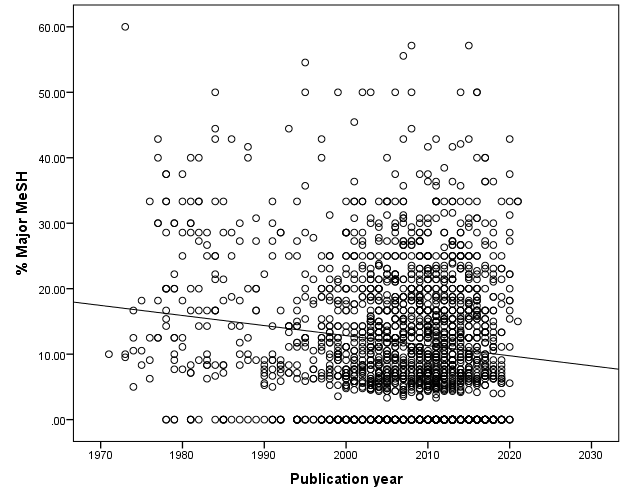


**S11. Sensitivity analyses - objectives declared in the 138 meta-analyses**

| **Id** | **PMID** | **Objective** | **Pharmacy** |
| --- | --- | --- | --- |
| 1 | 30538430 | The purpose of this review is to appraise the effect of **pharmacist-led educational interventions** on self-care activities and levels of glycated hemoglobin of T2DM patients. | clear |
| 2 | 30515250 | We aimed to systematically review the effectiveness of healthcare behavioral and education interventions for gout patients on clinical outcomes. | not |
| 3 | 30389320 | To systematically review the evidence that assessed the **outcomes of ward pharmacist** input for people admitted with acute or emergent illness. | clear |
| 4 | 30362920 | To assess the effect of a service containing self-management support **delivered by community pharmacists** to patients with asthma. | clear |
| 5 | 30319263 | We aimed to evaluate whether **pharmacist care** (PC) service model in LMIc and HIc could improve clinical outcomes in diabetic patients by performing a meta-analysis. | clear |
| 9 | 30178872 | To examine the effect of **pharmacists' non-dispensing services** on non-hospitalised patient outcomes. | clear |
| 13 | 29976652 | This systematic review and meta-analysis aims to evaluate how effective **pharmacist-led interventions** are in improving medication adherence in adults with asthma. | clear |
| 21 | 29692730 | This review aimed to evaluate and compare the efficacy of different **pharmacist based interventions** on clinical outcomes of type 2 diabetes patients. | clear |
| 37 | 29326185 | We determined whether interventions targeted at healthcare professionals are effective to enhance prescribing and health outcomes in patients with IHDs. | not |
| 38 | 29311916 | This review examines whether **pharmacist-led interventions** to support self-management in diabetes patients improve clinical and patient-reported outcomes. | clear |
| 39 | 29265170 | To examine the effect of interventions to optimize medication use on adverse drug reactions (ADRs) in older adults. | not |
| 40 | 29248878 | We systematically review the literature to evaluate intervention effectiveness in terms of discrepancy identification and resolution, clinical relevance of resolved discrepancies and healthcare utilisation, including readmission rates, emergency department attendance and primary care workload. | clear |
| 42 | 29224636 | The aim of this systematic review and meta-analysis was to evaluate the effects of **pharmacy-based interventions** on clinical outcomes associated with diabetes-related complications as well as on nonclinical outcomes in people with diabetes. | clear |
| 43 | 29145935 | We performed this systematic review and meta-analysis to confirm whether patients benefit more from **pharmacist-led** anticoagulation management than other models. | clear |
| 48 | 28948839 | To evaluate randomized controlled trials (RCTs) that included interventions **provided by community pharmacists** for patients with type 1 and 2 diabetes, the analysis of each component of the intervention(s), and the description of the training that the pharmacists received. | clear |
| 53 | 28851770 | Lay people or non-pharmacy health workers with training could dispense antiretroviral therapy (ART) in resource-constrained countries, freeing up time for pharmacists to focus on more technical tasks. We assessed the effectiveness of such task-shifting in low-income and middle-income countries. | not |
| 56 | 28671909 | The main objective of the current investigation was to examine the effectiveness of **pharmacist-based transition of care interventions** on the reduction of medication errors after hospital discharge. | clear |
| 58 | 28622997 | The aim of this review was to systematically evaluate the impact of **pharmacist interventions** on adherence to antidepressants and clinical symptomology among adult outpatients with depressive disorders. | clear |
| 61 | 28599601 | To describe **pharmacy-supported transition-of-care (TOC) interventions** and determine their effect on 30-day all-cause readmissions | clear |
| 63 | 28573873 | To conduct a comprehensive systematic review and meta-analyses examining the impact of **pharmacist interventions** as part of health care teams on diabetes therapeutic outcomes in ambulatory care settings. | clear |
| 70 | 28315760 | This systematic review applied meta-analytic procedures to integrate primary research that tested medication adherence interventions. | not |
| 75 | 28233442 | To compare the effectiveness of transitional care services in decreasing all-cause death and all-cause readmissions following hospitalization for heart failure (HF). | not |
| 80 | 28095780 | Therefore, the objective of this study is to assess the effectiveness of medication review as an isolated short-term intervention, irrespective of the patient population and the outcome measures used. | not |
| 84 | 28000212 | To assess the effects of interventions aimed at improving adherence to lipid-lowering drugs, focusing on measures of adherence and clinical outcomes. | not |
| 89 | 27873322 | To assess clinical, patient-reported, and resource use outcomes of non-medical prescribing for managing acute and chronic health conditions in primary and secondary care settings compared with medical prescribing (usual care). | not |
| 95 | 27696540 | This review sought to evaluate the effects of medication review on health-related quality of life (HRQoL) and healthcare costs in the elderly. | clear |
| 96 | 27677651 | The aim of the study is to compare the effectiveness of **pharmacist-managed** anticoagulation control of warfarin with other models. | clear |
| 97 | 27765379 | To complete a systematic review of the literature on the **impact of pharmacists** as educators, facilitators, and administrators of vaccines on immunization rates. | clear |
| 98 | 27566970 | To appraise the evidence critically for effectiveness of **pharmacy-based** needle/syringe exchange programmes (pharmacy-based NSPs) on risk behaviours (RBs), HIV/HCV prevalence and economic outcomes among people who inject drugs (PWID). | clear |
| 105 | 27511835 | The aim of this meta-analysis is to examine the impact of **in**-**hospital pharmacist-led** medication reviews in paediatric and adult patients. | clear |
| 108 | 27450138 | To estimate the impact that **pharmacist immunization programs** have on immunization rates. | clear |
| 109 | 27363846 | To evaluate the impact of **pharmaceutical care (PC) interventions** on health-related quality of life (HRQoL) and determine sensitivity of HRQoL measures to PC services. | clear |
| 128 | 26969094 | This systematic review applied meta-analytic procedures to integrate primary research that examined blood pressure outcomes of medication adherence interventions. | not |
| 131 | 26954666 | The interventions through **pharmacist-involved pharmaceutical care** in patients with heart failure (HF) and acute coronary syndrome (ACS) were reviewed systemically and examined. | clear |
| 133 | 26928025 | To systematically review the effectiveness of **community pharmacy-delivered interventions** for alcohol reduction, smoking cessation and weight management. | clear |
| 135 | 26913812 | This review sought to evaluate the impact of **pharmacy-led** medication reconciliation interventions on medication discrepancies at hospital transitions and to categorize these interventions as single transition interventions or multiple transitions interventions. | clear |
| 136 | 26908524 | Thus, the aim of this study was to systematically investigate the effect of **pharmacist-led** medication reconciliation programmes on clinical outcomes at hospital transitions. | clear |
| 159 | 26260916 | This systematic review focuses on controlled clinical trials evaluating the effect of **pharmacist intervention** on medication errors (MEs) in ICU settings. | clear |
| 168 | 25868941 | The aim of the present study was to conduct a meta-analysis of controlled trials assessing the impact of **pharmaceutical care interventions** (e.g. medication reviews) on medication underuse in older patients (≥65 years). | clear |
| 170 | 25810127 | Because pharmaceutical care might contribute to improved adherence to treatment, the aim of this study was to assess the impact of **pharmaceutical interventions** on ART via a systematic review of randomized clinical trials (RCT). | clear |
| 175 | 25581134 | Our objective was to synthesize the evidence on the effect of early **in-hospital pharmacist-led** medication review on patient-oriented outcomes based on observed data. | clear |
| 180 | 25401788 | To assess the effect of MTM interventions among outpatients with chronic illnesses. | not |
| 182 | 25330865 | The aim of this systematic review was therefore to assess  the influence of **pharmacist care** on health outcomes,  humanistic outcomes [e.g., quality of life (QoL), medication  compliance, and patient satisfaction], and healthcare  utilization in patients with COPD. | clear |
| 193 | 24966032 | To undertake a systematic review and meta-analysis of randomized controlled trials concerned with the impact of **community pharmacist-led** interventions on blood pressure control in patients with hypertension. | clear |
| 198 | 24749899 | The aim of this meta-analysis is to evaluate the effectiveness of smoking cessation interventions delivered by **community pharmacists** in assisting smokers to quit. | clear |
| 201 | 24721801 | By updating and combining data from 2 previous systematic reviews, we assess the effect of **pharmacist interventions** on BP and identify potential determinants of heterogeneity. | clear |
| 217 | 24196278 | This systematic review aimed to evaluate the effectiveness of interventions led by **hospital or community pharmacists** in reducing unplanned hospital admissions for older people. | clear |
| 218 | 24161491 | The aim of this study was to review the effectiveness of **clinical pharmacist services** delivered in primary care general practice clinics. | clear |
| 234 | 23796001 | To conduct a systematic review and meta-analyses to examine the effects of **pharmacists' care** on geriatric patient-oriented health outcomes in the United States (U.S.). | clear |
| 242 | 23594037 | The aim was to examine the impact of fee-for-service **pharmacist-led** medication review on patient outcomes and quantify this according to the type of review undertaken, e.g. adherence support and clinical medication review. | clear |
| 256 | 23173140 | This systematic review and meta-analysis of randomized controlled trials (RCTs) assesses the effect of **pharmacist care** on cardiovascular disease (CVD) risk factors among outpatients with diabetes. | clear |
| 279 | 21911628 | A systematic review was conducted to determine the impact of **pharmacist care** on the management of CVD risk factors among outpatients. | clear |
| 287 | 21610491 | We hypothesized that educational interventions delivered by **pharmacists** to patients with chronic pain might improve pain-related outcomes and sought to establish "proof of concept" for this hypothesis. | clear |
| 298 | 21258029 | **Pharmacist interventions** to enhance blood pressure (BP) control and adherence to antihypertensive therapy in adults with essential hypertension were reviewed. | clear |
| 299 | 21205952 | To systematically evaluate the effectiveness of **pharmacist** **care** in improving adherence of depressed outpatients to antidepressants. | clear |
| 308 | 20961643 | To conduct a meta-analysis evaluating the effect of **pharmacist intervention** on glycemic control. | clear |
| 309 | 20831620 | We performed a systematic review and meta-analysis to compare the effects of **PWTM [pharmacist-participated warfarin therapy management]** with usual care on bleeding and thromboembolic outcomes. | clear |
| 311 | 20720510 | The objective of this study was to conduct a comprehensive systematic review with focused meta-analyses to examine the effects of **pharmacist-provided** direct patient care on therapeutic, safety, and humanistic outcomes. | clear |
| 326 | 19858431 | The purpose of this systematic review was to determine the potency of interventions for BP **involving nurses or pharmacists.** | not |
| 341 | 18682540 | To quantify the impact of **pharmacist interventions** in enhancing patients' outcomes. | clear |
| 353 | 18093253 | We set out to determine the effects of **pharmacist-led** medication review in older people by means of a systematic review and meta-analysis covering 11 electronic databases. | clear |
| 357 | 17925496 | To identify and quantify outcomes sensitive to **pharmacists'** **interventions.** | clear |
| 359 | 17712043 | To identify outcomes sensitive to **pharmacists' interventions** and quantify their impact through critical literature review. | clear |
| 363 | 17420201 | A systematic review and metaanalysis were conducted to determine if studies that included **pharmacists** as chart reviewers detected higher rates of adverse drug events (ADEs) than studies that included other health care professionals or hospital personnel as chart reviewers. | clear |
| 371 | 16456206 | To identify and evaluate studies of interventions in primary care aimed at reducing medication related adverse events that result in morbidity, hospital admission, and/or mortality. | not |
| 483 | 26755524 | The primary objective of this review was to collate all the available evidence on the effectiveness of **pharmacist interventions** on the quality of prescribing among older hospitalised patients. | clear |
| 641 | 27660570 | The purpose of this metaanalysis was to evaluate the effectiveness of interventions aimed at improving adherence to lipid-lowering drugs, focusing on measures of adherence and clinical outcomes. | not |
| 679 |  | This study was undertaken to quantify and evaluate the impact of **pharmacist interventions** aimed at decreasing inappropriate SDL utilization. | clear |
| 865 | 28977687 | To determine the effectiveness of professional, organisational and structural interventions compared to standard care to reduce preventable medication errors by primary healthcare professionals that lead to hospital admissions, emergency department visits, and mortality in adults. | not |
| 916 |  | To evaluate the effect of **PC [Pharmaceutical Care]** programs on glycemic control in patients with diabetes mellitus. | clear |
| 1038 |  | To evaluate the effect of **pharmaceutical care (PC) programs** on blood pressure control in  individuals with hypertension. | clear |
| 1119 | 30175841 | To determine which interventions, alone or in combination, are effective in improving the appropriate use of polypharmacy and reducing medication-related problems in older people. | not |
| 1353 | 29693291 | A systematic review and meta-analysis of randomized controlled trials (RCTs) were performed to understand the effectiveness of medication adherence (MA) interventions among Chinese patients with hypertension. | not |
| 1377 | 26963251 | To assess the effect of **pharmacist interventions** on glycemic control in type 2 diabetic patients and to examine factors that could explain the variation across studies. | clear |
| 1409 | 29590146 | To update the previous assessment of **pharmacist-led** medication reconciliation by restricting the review to randomized controlled trials (RCTs) only. | clear |
| 1417 | 26560139 | This systematic review applied meta-analytic procedures to synthesize medication adherence interventions that focus on adults with hypertension. | not |
| 1421 | 29344366 | We conducted a systematic review and meta-analysis to determine the effectiveness of healthcare provider-led (HCPs) interventions to support medication adherence in patients with acute coronary syndrome (ACS). | not |
| 1472 | 27059768 | The aim of the present study was to explore the impact of strategies to reduce polypharmacy on mortality, hospitalization and change in number of drugs. | not |
| 1490 | 19155168 | To examine the effectiveness of multiple interventions as compared to single interventions or usual care on health outcomes and health care utilisation within the context of integrated disease management in asthma and COPD. | not |
| 1512 | 27549581 | This systematic review was thus, aimed to evaluate the impact of electronic medication reconciliation interventions on the occurrence of medication discrepancies at hospital transitions. | not |
| 1532 | 28417456 | To assess the efficacy and safety of interventions intended to improve adherence to inhaled corticosteroids among people with asthma. | not |
| 1540 | 27413005 | This review evaluated the effectiveness of interventions intended to improve adherence to statin medication. | not |
| 1551 | 27317347 | This article reports the results of a comprehensive systematic review and meta‐analysis of mortality and hospitalization outcomes from HF medication adherence (MA) intervention studies. | not |
| 1610 | 28483983 | The current meta-analysis and systematic review investigated whether adherence interventions improved immunosuppressive treatment adherence in kidney transplant recipients. | not |
| 2451 | 31097278 | We did a systematic review and meta-analysis of task-sharing interventions and their effects on managing blood pressure in LMICs. | not |
| 2495 | 30897055 | The aim of this study was to evaluate the impact of **pharmacist-led** discharge counseling on hospital readmission and emergency department visits through a systematic review and meta-analysis. | clear |
| 2527 | 31436877 | This study investigated the impact of **pharmacy-led** medication reconciliation on medication discrepancies and potential adverse drug events in the ED to assess the benefits of pharmacy services. | clear |
| 2568 | 30707465 | Our aim was to assess the benefit of **pharmacist delivered** educational interventions for patients with cancer pain. | clear |
| 2682 | 31135496 | The objective of this systematic review and meta-analysis was to assess the effects of including **critical care pharmacists** in multidisciplinary ICU teams on clinical outcomes including mortality, ICU length of stay, and adverse drug events. | clear |
| 2709 | 31427210 | To evaluate the impact of **community pharmacist** involvement on transitions of care, specifically on 30-day hospital readmissions. | clear |
| 2725 | 31102109 | A systematic review and meta-analysis were performed to determine the cumulative effect of **pharmacist-led** transitions of care on the 30-day all-cause readmission rates of patients with congestive heart failure with the objective to isolate and assess the effect of pharmacy intervention to a condition-specific service. | clear |
| 2747 | 31213852 | This systematic review aims to investigate the impact of collaborative practice between **community pharmacist** (CP) and general practitioner (GP) in asthma management. | clear |
| 2771 | 31351119 | The objective of this systematic review and meta-analysis  was to comprehensively evaluate the role of **pharmacist involved** multidisciplinary HF management, to determine  its effect in relation to HF hospitalizations, HF mortality,  all-cause hospitalizations, all-cause mortality, medication  adherence (compliance), HF knowledge, health-care costs,  self-care, and composite endpoint (all-cause hospitalizations,  all-cause mortality). | clear |
| 2786 | 30685443 | To review **pharmacist-led interventions** to improve medication adherence in patients with diabetes and to assess the effectiveness of these interventions on medication adherence. | clear |
| 2822 | 30698721 | To assess the effectiveness of ASPs **involving pharmacists** at improving antibiotic prescribing by general practitioners (GPs). | clear |
| 2852 | 31431337 | To perform a meta-analysis of **pharmacist and technician accuracy rates** when completing the final accuracy check on a prepared item. | clear |
| 2870 | 30920431 | The aim of the study was to assess the impact of medication reviews delivered by **community pharmacists** to elderly patients on polypharmacy. | clear |
| 2922 | 31225940 | Therefore, this study attempted to review the clinical outcomes of CPS. Interventions included are provision of medication review, patient education, adherence assessment, health/lifestyle advice, physical assessment, monitoring, prescribing, or adjusting and administering therapy **from community pharmacists**. | clear |
| 2933 |  | To evaluate the effect of **pharmaceutical care** (PC) on glycemic control in  patients with type 2 diabetes mellitus. | clear |
| 3003 | 32419932 | To clarify the role of the **clinical pharmacist consultation** **service** in the management of infectious diseases (ID). | clear |
| 2382 | 31941489 | To determine the effectiveness of **pharmacist home visits** for individuals at risk of medication-related problems we undertook a systematic review and meta-analysis of randomised controlled trials (RCTs). | clear |
| 2386 | 31777082 | To conduct a systematic review and meta-analysis of the effectiveness of general practice-based **pharmacist interventions** in reducing the medical risk factors for the primary prevention of cardiovascular events. | clear |
| 2415 | 31868236 | To examine the effects of **pharmacy-based management** **interventions** compared with active control (e.g. patient information materials or any other active intervention delivered by someone other than the pharmacist or the pharmacy team), waiting list, or treatment as usual (e.g. standard pharmacist advice or antidepressant education, signposting to support available in primary care services, brief medication counselling, and/or (self-)monitoring of medication adherence offered by a healthcare professional outside the pharmacy team) at improving depression outcomes in adults. | clear |
| 2433 | 31684695 | To assess the effectiveness of **interventions delivered by community pharmacy personnel** to assist people to stop smoking, with or without concurrent use of pharmacotherapy. | clear |
| 2390 | 31465121 | As such, the aim of our current systematic review was to provide an overview of the evidence of **pharmacist‐led** interventions to improve the quality use of medicine in nursing homes and determine the impact of these interventions in nursing homes. | clear |
| 2444 | 31711390 | The main objective of this meta-analysis was to analyze the impact of **pharmacist-led** MRs on cardiovascular disease risk factors overall and in different ambulatory settings while exploring the effects of different components of MRs. | clear |
| 2443 | 31758656 | To analyze the impact of clinical medication reviews (CMR) on reducing unplanned hospitalizations owing to polypharmacy among older adults using an intervention. | not |
| 2384 | 31919801 | Our objective was to systematically review interventions to reduce the incidence of ADEs measured by health outcomes in older patients in primary care settings. | not |
| 10135 | 32418821 | The objective of the study was to conduct a meta-analysis to determine the effectiveness of **pharmacist interventions** on reducing LDL-C levels. | clear |
| 10147 | 32107837 | The objective of our study is to evaluate the effect of **pharmacist-led** interventions on asthma and COPD management, focusing mainly on inhalation technique and medication adherence, and whether the content of interventions (categorized based on Information-Motivation-Behavioural skills (IMB) model) affects the effectiveness and whether the IMB model is worthy of clinical promotion and application in adults with asthma or COPD. | clear |
| 10125 | 32487066 | In the present study, a systematic review and meta-analysis for pooling statistical power was conducted to systematically evaluate the clinical and economic outcomes of **hospital pharmaceutical care.** | clear |
| 10095 | 33240478 | The objective of this review was to compare the effectiveness of different interventions in reducing prescribing, dispensing and administration medication errors in acute medical and surgical settings. | not |
| 10139 | 32328958 | To qualitatively and quantitatively evaluate the impact of **clinical pharmacist interventions** on medication error rates for hospitalized pediatric patients. | clear |
| 10092 | 33294063 | To map the **clinical pharmacy services** conducted in Brazil, their characteristics, outcomes, and process measures in general population, as well as the assessment of the clinical impact on people with cardiometabolic diseases (cardiovascular diseases and metabolic diseases). | clear |
| 10154 | 32022107 | To investigate the impact of **pharmaceutical care-based** **interventions** on type 2 diabetes mellitus . | clear |
| 10149 | 32078109 | To investigate the impact of **clinical pharmacist services** on patients' length of hospitalization, readmission and mortality in China. | clear |
| 10093 | 33270710 | Conduct a systematic review and meta-analysis to estimate the impact of **pharmacy-supported interventions** on the proportion of patients discharged from the hospital on inappropriate acid suppressive therapy (AST). | clear |
| 10121 | 32629653 | To explore the **role of pharmacists and impact of pharmacy** **interventions** for PD patients. | clear |
| 10023 | 34353754 | This study aims to determine the effects of **pharmacist intervention** on improving adherence to antiretroviral therapy (ART), viral load (VL) suppression, and change in CD4-T lymphocytes in PLWHA. | clear |
| 10046 | 33965357 | To evaluate the effectiveness of **community-pharmacist-based** medication review programmes among patients with long-term conditions. | clear |
| 10713 |  | Therefore, we performed an  updated systematic review and meta-analysis of RCTs evaluating the  effects of **pharmacist intervention** on patients with HF. | clear |
| 10017 | 34459265 | To systematically review and analyze randomized controlled trials assessing the impact of **pharmacist services** on patients with depression compared to usual care using a meta-analysis approach. | clear |
| 10761 | 28671909 | The main objective of the current investigation was to examine the effectiveness of **pharmacist-based** transition of care interventions on the reduction of medication errors after hospital discharge. | clear |
| 10027 | 34303610 | To assess the impact of **pharmacist-led interventions**, which include communication with a primary care physician (PCP) on reducing hospital readmissions. | clear |
| 11005 | 34228962 | This study aimed to systematically investigate the effect of **multidisciplinary teams that include coordinated pharmaceutical care** on clinical outcomes. | clear |
| 10033 | 34161388 | To describe and compare various **pharmacist-led** educational interventions delivered to healthcare providers and to evaluate their impact qualitatively and quantitatively on medication error rates. | clear |
| 10063 | 33678564 | To determine the association of **pharmacist** medication counseling with medication adherence, 30-day hospital readmission, and mortality. | clear |
| 10008 | 34716722 | Hence, this systematic review and meta-analysis was aimed at investigating **pharmacists' functions** in ASPs in critically ill neonates and the effect of ASP implementation on antibiotic use. | clear |
| 10054 | 33817821 | This study aimed to evaluate the effects of **pharmacists'** educational interventions in the **community pharmacy** settings on asthma control and severity, quality of life (QOL) and medication adherence. | clear |
| 10029 | 34287846 | The objective was to assess the effectiveness of **pharmacist-led interventions** on medication adherence in older adults (65+ years). | clear |
| 10844 |  | For this reason, our study aims to evaluate the intervention  of the **pharmacist and pharmacist-physician**  **cooperation** by meta-analyses and interprets the effectiveness  of these interventions in view of all available  literature. | clear |
| 10040 | 34047881 | The aim of this study was to identify key success factors for effective **pharmacy intervention** design and implementation to improve vaccination acceptance rates in influenza. | clear |
| 10105 | 32949161 | In this study, we aimed to assess the effectiveness of **pharmacist intervention** on BP control in patients with CKD and evaluate the usefulness of home-based BP telemonitoring. | clear |
| 10109 | 32881191 | To conduct a systematic review of the impact of **community pharmacist interventions** on patient adherence to lipid lowering medication (LLM) prescriptions and clinical outcomes. | clear |
| 10032 | 34180129 | To systematically investigate the effect of interventions to overcome therapeutic inertia on glycaemic control in individuals with type 2 diabetes. | not |
| 10030 | 34240570 | Because most HF is managed outside of hospitals, we aimed to synthesize the evidence for **pharmacist care** in outpatients with HF. | clear |
| 10076 | 33486825 | The current review aims to comprehensively summarize  **pharmacist-led** intervention on pain management, either individually  or in a multidisciplinary team, irrespective of setting. | clear |
| 10003 | 34848531 | This meta-analysis aims to evaluate inappropriate antibiotic prescribing in the Gulf region and determine the effect of **pharmacist-led** antimicrobial stewardship (AMS) programmes on reducing inappropriateness. | clear |

**S12. Social network analyses of co-occurrence of any MeSH term assigned to the articles**

Yellow nodes represent the MeSH terms of the ‘Pharmaceutical Services’ branch. Green nodes represent pharmacy-specific MeSH terms. Blue nodes represent the remaining, not pharmacy-specific MeSH terms.

**S13. Social network analyses of co-occurrence of Major MeSH term assigned to the articles**

Yellow nodes represent the MeSH terms of the ‘Pharmaceutical Services’ branch. Green nodes represent pharmacy-specific MeSH terms. Blue nodes represent the remaining, not pharmacy-specific MeSH terms.
